# Genome-Wide Detection and Analysis of Multifunctional Genes

**DOI:** 10.1371/journal.pcbi.1004467

**Published:** 2015-10-05

**Authors:** Yuri Pritykin, Dario Ghersi, Mona Singh

**Affiliations:** 1 Department of Computer Science, Princeton University, Princeton, New Jersey, United States of America; 2 Lewis–Sigler Institute for Integrative Genomics, Princeton University, Princeton, New Jersey, United States of America; 3 School of Interdisciplinary Informatics, University of Nebraska at Omaha, Omaha, Nebraska, United States of America; Tufts University, UNITED STATES

## Abstract

Many genes can play a role in multiple biological processes or molecular functions. Identifying multifunctional genes at the genome-wide level and studying their properties can shed light upon the complexity of molecular events that underpin cellular functioning, thereby leading to a better understanding of the functional landscape of the cell. However, to date, genome-wide analysis of multifunctional genes (and the proteins they encode) has been limited. Here we introduce a computational approach that uses known functional annotations to extract genes playing a role in at least two distinct biological processes. We leverage functional genomics data sets for three organisms—*H. sapiens*, *D. melanogaster*, and *S. cerevisiae*—and show that, as compared to other annotated genes, genes involved in multiple biological processes possess distinct physicochemical properties, are more broadly expressed, tend to be more central in protein interaction networks, tend to be more evolutionarily conserved, and are more likely to be essential. We also find that multifunctional genes are significantly more likely to be involved in human disorders. These same features also hold when multifunctionality is defined with respect to molecular functions instead of biological processes. Our analysis uncovers key features about multifunctional genes, and is a step towards a better genome-wide understanding of gene multifunctionality.

## Author Summary

Almost every aspect of cellular function depends on protein activity. In spite of being fine-tuned to carry out highly specific functions, proteins can also multitask. Experimental studies have identified genes and proteins endowed with more than one molecular function, or participating in very different biological processes. These studies suggest that the degree of functional plasticity exhibited by proteins might go well beyond a simple “one protein—one function” relationship. However, systematic studies of the properties of multifunctional genes (and their encoded proteins) have been limited. Here we present a computational framework to identify putative multifunctional genes, and compare their properties with those of other genes. We find that multifunctional genes are significantly different from other genes with respect to their physicochemical properties, expression profiles, and interaction properties. We also observe that multifunctional genes tend to be more conserved, and that a greater fraction of them are associated with human disorders. Taken together, these results represent a step towards a more complete understanding of the role multifunctional genes play in the functional organization of the cell.

## Introduction

Multifunctionality can be defined as the involvement of a gene in multiple cellular processes [1]. This can come about either because a protein coded by a gene is capable of performing distinct molecular functions [2–6], or as a result of a single molecular function being performed in different contexts [7, 8]. For example, pioneering experimental work led to the surprising finding that crystallins—the proteins responsible for the optical properties of the eye lens—can also play non-refractive roles and have enzymatic activity in other tissues [2]. This evolutionary strategy was named “gene sharing” [9]. Further examples of proteins performing multiple molecular functions were subsequently described: a uracil-DNA glycosylase that can also function as a glyceraldehyde-3-phosphate dehydrogenase, or the enzyme thrombin that can moonlight as a ligand for surface receptors [3]. More recently, a large-scale screening of mutants in yeast was performed to measure the pleiotropic effects of genes under different conditions [10]. In the case of pleiotropy, a gene may perform only one molecular function, but it can be involved in multiple biological processes, and its perturbation can therefore have pleiotropic consequences.

Though multifunctionality has been characterized in detail only for a few case studies, it is likely to be a common phenomenon. Nevertheless, multifunctionality remains poorly understood. Fortunately, the current state of known gene functional annotations for several organisms gives us an opportunity to systematically identify multifunctional genes and analyze their properties. Earlier computational studies have attempted to identify multifunctional genes from functional annotations available for genes in different organisms. Several previous works measured multifunctionality by simply counting the number of distinct Gene Ontology (GO) biological process terms annotating a gene product [11–14]. While intuitive and straightforward, these approaches do not always guarantee that a gene annotated with more than one GO term is indeed involved in two distinct biological processes. In particular, this assumption is incorrect when one term is a descendant of another term in the GO hierarchy. To better handle the hierarchical organization of GO, an alternate approach considered the total number of distinct GO “leaf” terms annotating a gene [15], and a recent analysis used semantic similarity between GO terms to identify moonlighting proteins [16]. However, problems may also arise even when two terms are in completely different branches of the ontology, as idiosyncrasies in GO may lead to similar processes being categorized in distinct places in the ontology. Methods to overcome this redundancy by focusing on a manually curated subset of terms (e.g., GO Slim or other gold standards [17–19]), even though suitable for tasks such as function prediction, can introduce a bias from manual curation to the analysis of gene multifunctionality, and also may not be generalizable as more annotations become available. Other approaches have used protein-protein interaction data and defined proteins as multifunctional if they are located at the intersection of overlapping clusters [20]. However, computationally derived clusters can differ substantially depending on the algorithm used [21], thereby leading to imprecise views of multifunctionality. Further, using interaction data to define multifunctional genes has the obvious drawback of preventing an unbiased analysis of these genes’ network properties.

In our work, we develop a computational approach to identify multifunctional genes that leverages GO functional annotations in a systematic and robust manner. To handle similar terms that appear in distant places in GO, we explicitly select sets of terms that do not co-annotate an enriched number of genes; these terms are then used to identify multifunctional genes. We apply our procedure to detect multifunctional genes to three organisms—human, fly and yeast—and then compare in each organism the properties of multifunctional genes (and the proteins they encode) with those of other annotated genes. Our results across these species consistently show that, as compared to other genes, multifunctional genes possess distinct physicochemical properties, are more broadly expressed across cell types and tissues, tend to be more evolutionarily conserved, are more likely to be essential, and are topologically distinct in protein-protein interaction networks, in regulatory transcription factor–gene networks and in genetic interaction networks. We also find that multifunctional genes are significantly more likely to be involved in human disorders than other genes. Overall, our analysis leads to a more complete understanding of the role multifunctional genes play in the functional organization of the cell.

## Results

### Genome-wide detection of multifunctional genes

**Fig 1:**
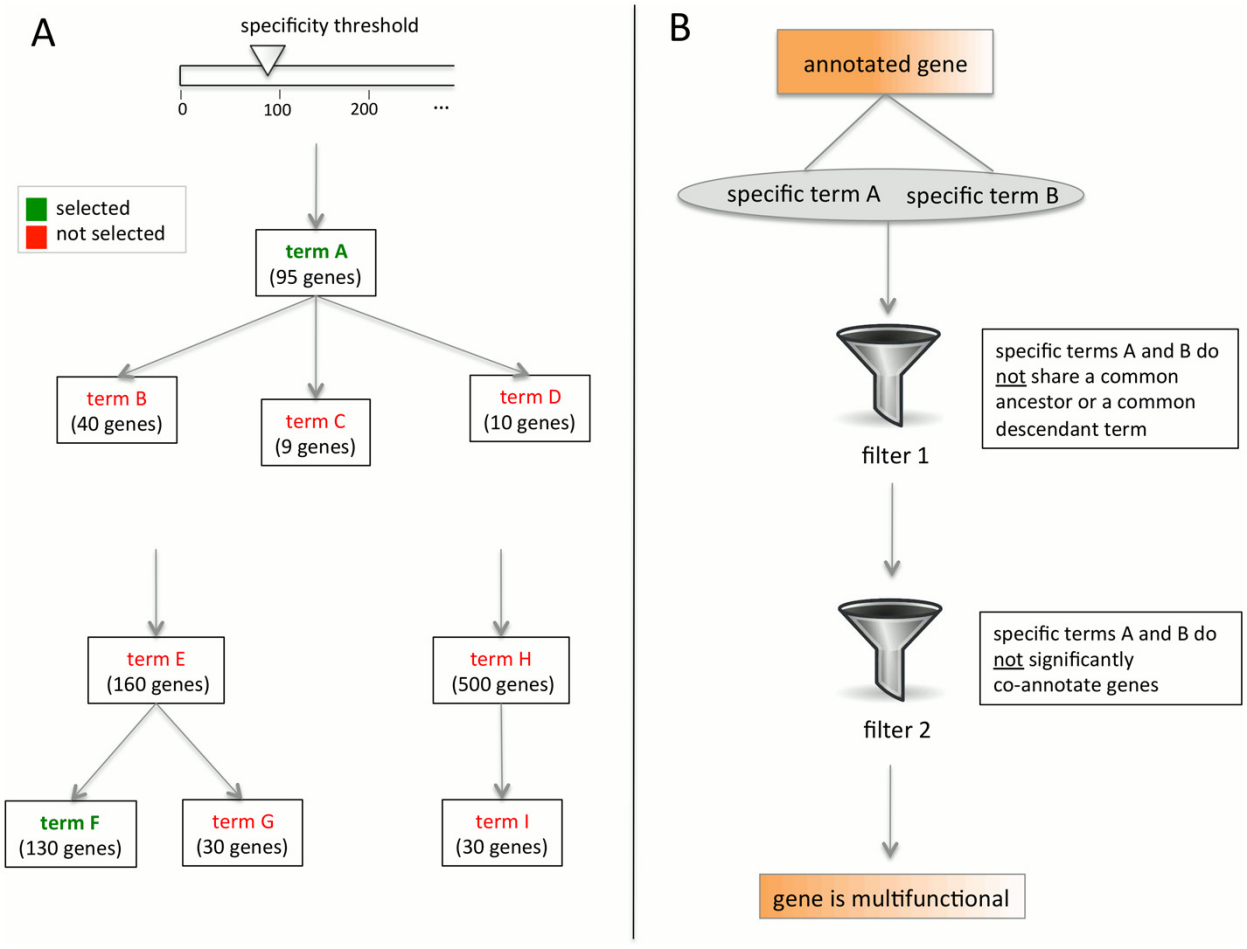
Schematic representation of the pipeline to identify multifunctional genes. We define as multifunctional all genes that have two or more annotations by distinct terms of comparable specificity. (A) First, we extract a subset of Gene Ontology terms at a comparable level of specificity. For a specificity threshold *N*, we select all terms that annotate at least *N*, but fewer than 2*N* genes, whose every descendant term (if any) annotates fewer than *N* genes. For example, if *N* = 90, then terms A and F are selected because each of them annotates more than 90 genes and less than 180 genes, and each of their descendant terms annotates less than 90 genes. In contrast, term E is rejected, because its descendant term F annotates more than 90 genes. Term H is also rejected, because it annotates more than 180 genes. (B) Once terms at a certain specificity level have been selected, we extract all genes annotated with at least two such terms. In order to consider annotations by distinct terms only, from the collection of all pairs of terms selected at the chosen level of specificity, we filter out those that either share a common ancestor (other than the root) or have a common descendant term in the GO graph. Further, we remove all pairs of terms that co-annotate more genes than expected by chance, as measured by the hypergeometric test. All genes co-annotated by some pair of terms (chosen at any considered level of specificity) passing these two filters are considered multifunctional.

We use functional annotations of genes in three organisms, *H. sapiens*, *D. melanogaster*, and *S. cerevisiae*, to identify multifunctional genes in each of them at the genome-wide level. To accomplish this, we use Biological Process GO annotations [22], though in subsequent analyses we also consider multifunctionality with respect to Molecular Function. In the remaining text, when we refer to GO annotations, we refer to Biological Process terms unless otherwise specified. Our method for detecting multifunctional genes is shown schematically in Fig 1 and is briefly described below (see Materials and Methods for details).

The Biological Process GO is a hierarchy of terms representing different aspects of biological processes, where the terms range from very general to very specific and a relationship between terms indicates if one term implies another. We therefore start by selecting a subset of comparable terms that do not have ancestor or descendant relationships amongst themselves. This set of terms can be chosen at different specificity levels, represented by a parameter *N* corresponding to the number of genes annotated by a term. Lower values of this parameter produce larger numbers of more specific terms, and higher values result in smaller numbers of more general terms (S1 Fig). We consider several distinct levels of specificity and call multifunctional all genes for which we find evidence of multifunctionality at any specificity level.

Once the terms have been selected at a particular specificity level, we extract all genes annotated with at least two such terms. In order to select only pairs of distinct terms and make sure a gene annotated by both terms is truly multifunctional, we apply several filters to pairs of terms. From the collection of all pairs of terms at a particular specificity level, we filter out those that either share a common ancestor (other than the root) or have a common descendant term in the GO graph, as these events indicate that the terms are semantically related. However, this is not sufficient to claim that the remaining pairs of terms are distinct. For example, the terms aerobic respiration and mitochondrial translation do not have any ancestral or descendant term in common in the GO hierarchy graph besides the most general biological process term, but often co-annotate mitochondrial ribosomal proteins and capture semantically distinct aspects of the same function. Therefore, we further remove all pairs of terms that co-annotate more genes than expected by chance (as detected by the hypergeometric test). All genes co-annotated by some pair of chosen terms passing these two filters, for any set of chosen terms at each specificity level *N* considered, are called multifunctional. We note that, depending upon the application, our filters can be relaxed to consider more genes as multifunctional; for example, two biological processes may be considered distinct if they share a common ancestor that is sufficiently general. However, here we aim to identify genes that have the strongest evidence of multifunctionality.

In what follows, we compare multifunctional genes detected in fly, human, and yeast with all other annotated genes in these organisms in order to uncover whether there are significant differences between the two groups with respect to various biological properties. The number of multifunctional genes and the total number of annotated genes for each organism is given in Table 1, and the actual lists of identified multifunctional genes are provided as S1 File for fly, S2 File for human, and S3 File for yeast. We note that a small number of experimentally verified human, fly and yeast genes with multiple functions are known [23, 24], and our method is able to successfully detect a significant fraction of these genes (see Section 1.1 in S1 Text).

**Table 1:** Number of multifunctional genes.

Organism	Number of multifunctional genes detected	Total number of annotated genes
*D. melanogaster*	1509	6354
*H. sapiens*	2517	9664
*S. cerevisiae*	876	4682

For each organism, we show the number of multifunctional genes detected by our method and the total number of annotated genes (annotated by one of the terms used to detect multifunctionality; see Fig 1 and Materials and Methods).

### Proteins encoded by multifunctional genes are longer, have more domains and have a higher fraction of disordered residues

**Fig 2:**
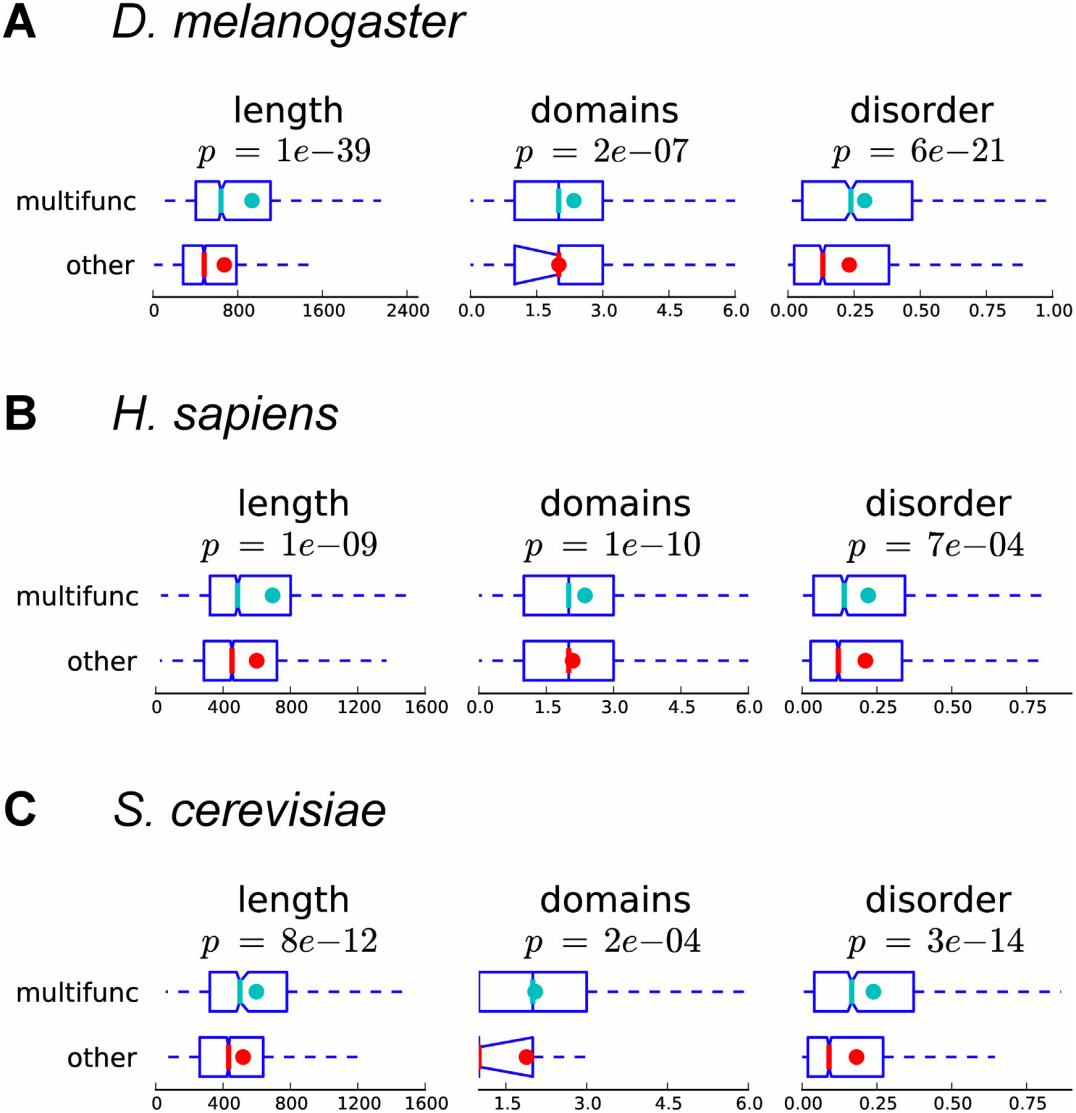
Proteins encoded by multifunctional genes are longer, have more domains, and are more disordered. Boxplots for length, number of unique domains, and fraction of disordered residues in proteins encoded by multifunctional and other annotated genes are shown for (A) fly, (B) human, and (C) yeast. Colored dots show the means, notches show bootstrap-generated 95% confidence intervals around the medians, boxes show quartile ranges, and whiskers extend to the most extreme data points within 1.5 times the size of the inner quartile range. For genes in fly and human, if a gene has more than one protein isoform, the longest isoform is considered. Multifunctional genes are significantly longer, have a significantly larger number of unique domains, and are significantly more disordered (Mann–Whitney U test).

We start the analysis by studying some basic physicochemical properties of proteins. First, we hypothesized that multifunctional proteins may be longer than other proteins in order to accommodate more functional domains. To test this hypothesis, we compare the lengths of proteins encoded by multifunctional and other annotated genes in *D. melanogaster*, *H. sapiens*, and *S. cerevisiae*, and indeed find that multifunctional genes are significantly longer than other genes (*p*-values 1*e*-39, 1*e*-9, and 8*e*-12, respectively, Mann–Whitney U test), on average by 39%, 16%, and 15%, respectively (Fig 2). We also observe that proteins encoded by multifunctional genes have significantly higher numbers of distinct domains per protein (*p*-values 2*e*-7, 1*e*-10, and 2*e*-4, respectively), on average by 17%, 13%, and 8%, respectively (Fig 2); this is consistent with the earlier finding of a small but statistically significant positive correlation between the number of GO biological process leaf terms a gene has and its number of Pfam domains [15]. However, we also note that longer proteins have more domains, so the difference in length between multifunctional and other genes can potentially explain the observed difference in the number of domains (see Section 1.2 in S1 Text).

Another mechanism that has been proposed to play a role in protein multifunctionality is the presence of intrinsically unstructured regions, which are thought to increase the structural adaptability of interaction surfaces of proteins to allow them to bind to the same or distinct partners with different effects [25]. To determine whether multifunctional proteins tend to be more disordered, we predict the fraction of disordered residues using the IUPred program [26, 27], and find that multifunctional genes in *D. melanogaster*, *H. sapiens*, and *S. cerevisiae* have a significantly higher fraction of predicted disordered residues (*p*-values 6*e*-21, 7*e*-4, and 3*e*-14, respectively), on average by 26%, 5%, and 31%, respectively (Fig 2). These results are in agreement with recent analyses of disordered regions in experimentally verified moonlighting proteins and a small set of computationally inferred moonlighting proteins in *E. coli* [16].

Overall, we find that proteins encoded by multifunctional genes are longer, have more domains and are more disordered than proteins encoded by other annotated genes.

### Multifunctional genes are expressed more broadly in fly and human

**Fig 3:**
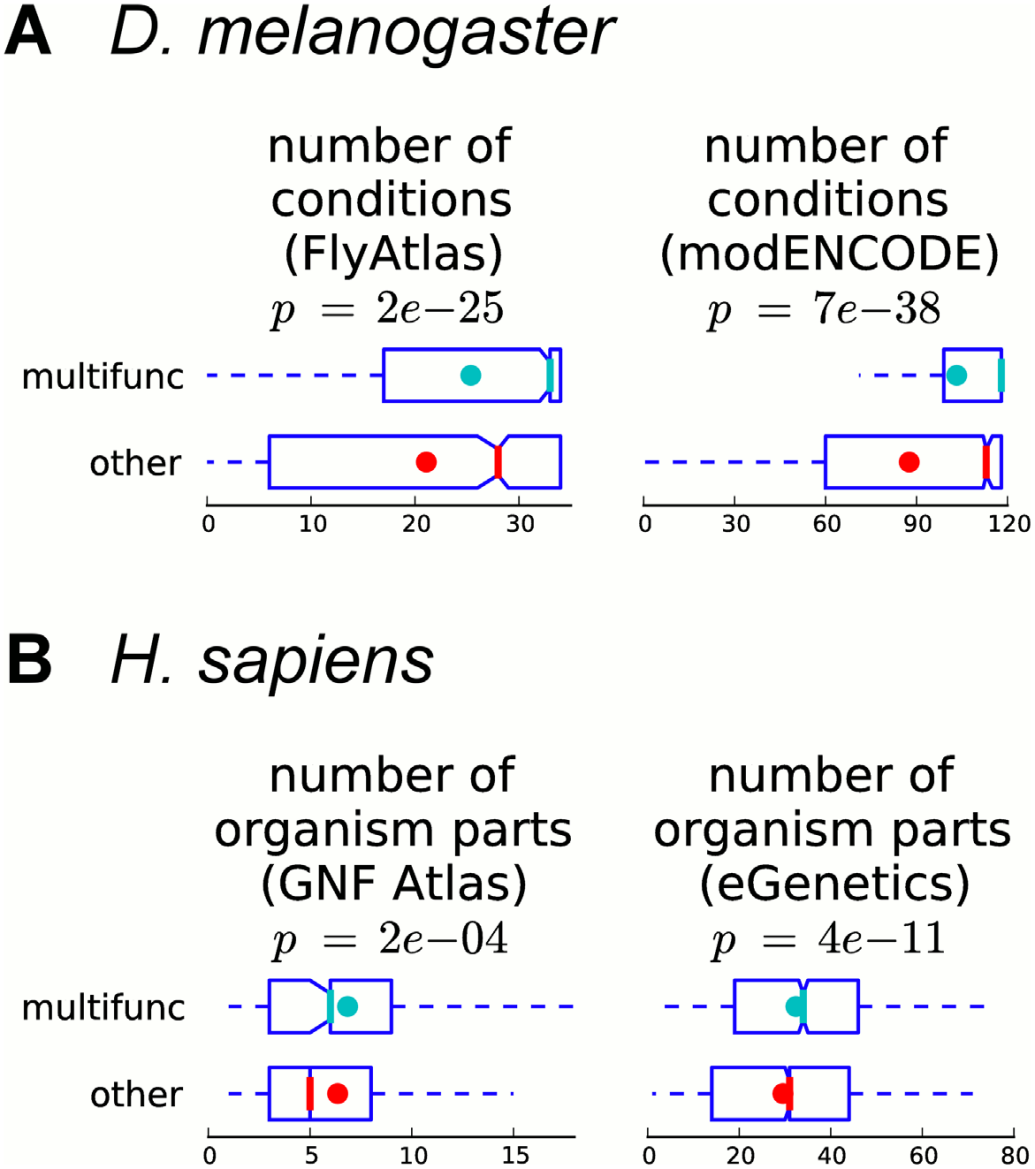
Multifunctional genes are more broadly expressed. Boxplots of the number of organism parts and/or conditions in which multifunctional and other annotated genes are expressed are shown for (A) fly (microarray expression data from FlyAtlas and RNA-seq expression data from modENCODE) and (B) human (GNF atlas and eGenetics expression data obtained from Ensembl). Colored dots show the means, notches show bootstrap-generated 95% confidence intervals around the medians, boxes show quartile ranges, and whiskers extend to the most extreme data points within 1.5 times the size of the inner quartile range. Multifunctional genes are expressed in a significantly larger number of conditions than other annotated genes (Mann–Whitney U test).

Differential gene expression is evident across tissues and cell types. A gene expressed in different contexts may have different functions depending upon how and when it is expressed. Therefore, we hypothesized that a gene associated with multiple distinct functions may be expressed in a larger number of contexts. In order to assess the relationship between gene expression and gene multifunctionality, we use genome-wide mRNA expression data and count in how many conditions, tissues or cell types each gene is expressed. For fly, we use two datasets: (1) FlyAtlas [28], the Drosophila microarray gene expression atlas across different tissues in larva and adult, and (2) RNA-seq data from modENCODE across many different tissues and development time points, as aggregated by FlyBase [29, 30]. For human, we use information about organism parts in which genes are expressed, obtained from Ensembl BioMart [31]. We observe that in both human and fly, multifunctional genes are expressed more broadly than other annotated genes; that is, they are expressed in a significantly larger number of tissues or organism parts (*p*-values from 7*e*-38 to 2*e*-4, Mann–Whitney U test; Fig 3A and 3B).

A potential mechanism for gene multifunctionality is the production via alternative splicing of multiple protein isoforms with different functions. Indeed, we observe that multifunctional genes have a significantly larger number of known isoforms in fly and human (S2 Fig). If different isoforms of a gene have different expression patterns, this gene may be detected as broadly expressed in genome-wide assays, which currently report expression only at the gene level, merging information about the expression of different isoforms. Indeed, we observe a significant positive correlation between the number of isoforms per gene and the number of tissues or organism parts in which it is expressed (S1 Table). However, when comparing genes with an equal number of known isoforms, we still observe that multifunctional genes are expressed in larger numbers of tissues or organism parts (although most *p*-values for human are above our significance threshold of 5%; S2 Fig). This indicates that multifunctional genes are more broadly expressed regardless of the number of isoforms.

### Multifunctionality is evolutionarily conserved

**Fig 4:**
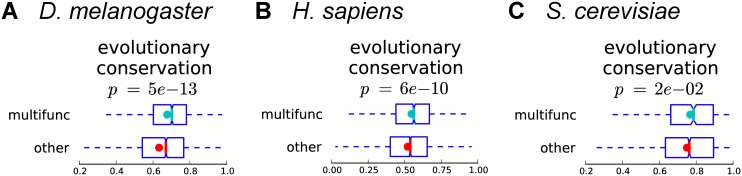
Multifunctional genes are more evolutionarily conserved. Boxplots of evolutionary conservation (estimated by phastCons [32] for each nucleotide, averaged over the nucleotides of each gene) of multifunctional and other annotated genes are shown for (A) fly, (B) human, and (C) yeast. Colored dots show the means, notches show bootstrap-generated 95% confidence intervals around the medians, boxes show quartile ranges, and whiskers extend to the most extreme data points within 1.5 times the size of the inner quartile range. Multifunctional genes are significantly more evolutionary conserved than other annotated genes (Mann–Whitney U test).

Acquiring multiple functions may constitute a special evolutionary strategy and limit gene evolutionary rates [9]. In order to study the evolutionary dynamics of gene multifunctionality at the genome-wide level and in an unbiased manner, we use evolutionary conservation scores from phastCons [32]. Scores in phastCons are computed using phylogenetic hidden Markov models of multiple sequence alignments of *D. melanogaster* with 14 other insect genomes, of *H. sapiens* with 99 other vertebrate genomes, and of *S. cerevisiae* with 6 other yeast species. For each nucleotide of the genome, phastCons produces a score between 0 and 1, where higher values indicate stronger evolutionary conservation. For each gene, we average the scores of all nucleotides of each isoform of the gene, and then average over all isoforms of the gene to obtain a single value for each gene as an estimate of how evolutionarily conserved the gene is. Previously, a positive correlation between the number of biological process GO terms a protein is annotated with and its evolutionary conservation was observed for yeast [7, 11, 33]. In agreement with this, we find that in fly, human, and yeast, multifunctional genes are significantly more evolutionarily conserved than other annotated genes (*p*-values 5*e*-13, 6*e*-10 and 0.02, respectively, Mann–Whitney U test; Fig 4).

Having shown that multifunctional genes tend to evolve more slowly, we next hypothesized that multifunctional genes independently detected in different organisms may be orthologous to each other. In order to test this, we compare the property of multifunctionality for orthologous proteins from different organisms. We use information about protein orthology from P-POD [34] and count how many orthologous pairs are observed where both corresponding genes are identified as multifunctional. Between fly and human, we observe 1725 orthologous pairs of genes where one gene is classified as multifunctional in fly and the other gene is classified as multifunctional in human. To assess significance, we compute the same number when randomly reshuffling multifunctional and other annotated genes from orthologous pairs in each organism, and observe on average only 845.1 ± 90.0 orthologous pairs where both genes are classified as multifunctional; thus, the actual value is 2.0 times higher (empirical *p*-value < 1*e*-3). For fly and yeast, we find 388 orthologous pairs between multifunctional genes (2.1 times higher than 184.7 ± 20.2 expected by chance, *p* < 1*e*-3). For human and yeast, we find 576 orthologous pairs between multifunctional genes (2.2 times higher than 267.2 ± 32.6 expected by chance, *p* < 1*e*-3). We conclude that the property of multifunctionality is conserved across orthologous genes of different organisms. This observation also supports the validity of our method for detecting multifunctional genes.

Functional annotations of genes are in part determined by transferring information between organisms via sequence similarity, and this could potentially confound our evolutionary analysis of multifunctionality. To address this, we repeat the analysis excluding GO annotations based on sequence or structural similarity and observe the same trends (see Section S1.3 in S1 Text and S14 Fig).

### Multifunctional genes are involved in more regulatory and genetic interactions

**Fig 5:**
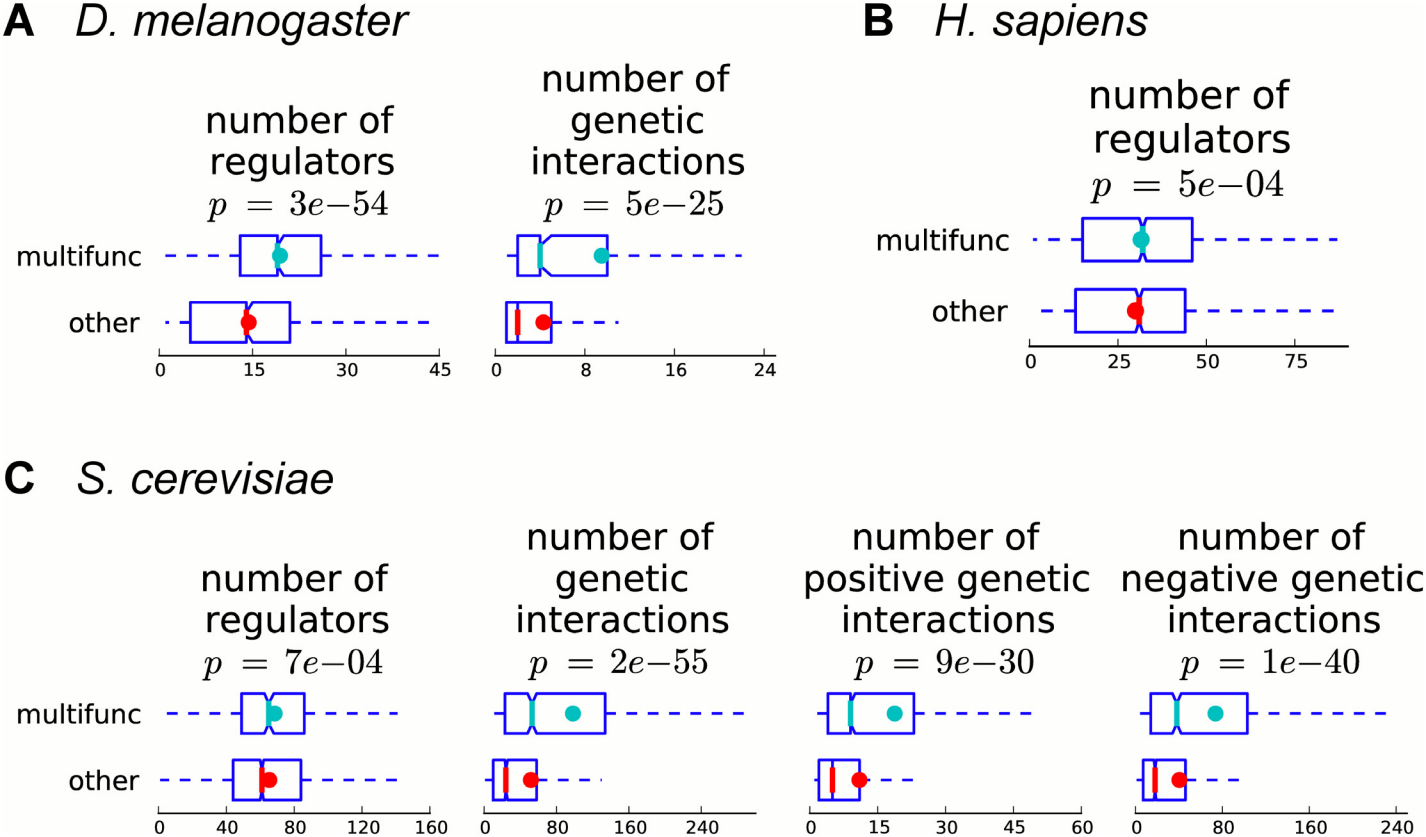
Multifunctional genes are involved in a significantly larger number of regulatory and genetic interactions. Boxplots of the number of regulatory and/or genetic interactions for multifunctional and other annotated genes are shown for (A) fly, (B) human, and (C) yeast. Colored dots show the means, notches show bootstrap-generated 95% confidence intervals around the medians, boxes show quartile ranges, and whiskers extend to the most extreme data points within 1.5 times the size of the inner quartile range. Multifunctional genes are involved in significantly more regulatory and genetic interactions (Mann–Whitney U test).

Genes responsible for multiple functions may require more complex regulatory programs to differentiate functions across multiple tissues or conditions. In order to study how regulated multifunctional genes are, we use regulatory interactions from high-throughput ChIP experiments [35–38]. For each gene, we count the number of transcription factor–target interactions this gene participates in as a target. In all three organisms, we observe that multifunctional genes are regulated by a significantly larger number of transcription factors than are other annotated genes (*p*-values from 3*e*-54 to 7*e*-4, Mann–Whitney U test; Fig 5).

In addition to requiring more complex regulatory programs, multifunctional genes may also be associated with more complex phenotypes that require involvement with many other genes; this would be reflected in a gene’s genetic interactions. In order to compare the distribution of genetic interactions between multifunctional and other annotated genes, we use a collection of genetic interactions curated by FlyBase [30] for fly and by BioGRID [39] for yeast. Previously, a positive correlation between the number of biological process GO annotations a gene has and its number of genetic interactions was observed for yeast [19]. In agreement with this, we observe that in fly and yeast, the number of genetic interactions is significantly higher for multifunctional genes than for all other annotated genes (*p*-values 5*e*-25 and 2*e*-55, respectively; Fig 5). Moreover, in a more refined comparison for yeast, we observe that both the number of positive and the number of negative genetic interactions are significantly larger for multifunctional genes than for other annotated genes (*p*-values 9*e*-30 and 1*e*-40, respectively; Fig 5).

### Multifunctional genes are more often essential

A gene associated with multiple functions may be more important for the normal functioning of the cell and therefore may potentially be more critical for survival than a gene associated with a single function. In order to test this hypothesis, we consider the relationship between gene essentiality and multifunctionality.

**Fig 6:**
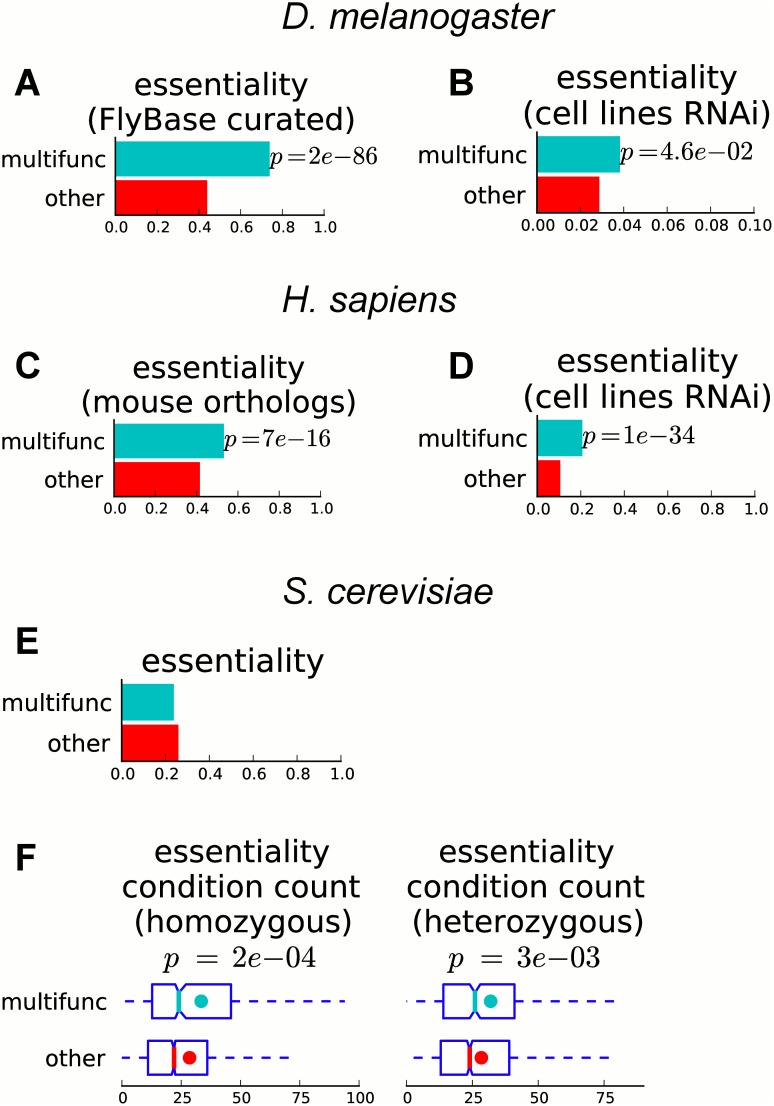
Multifunctional genes are more likely to be essential. Barplots showing the fraction of multifunctional and other annotated genes that are essential in (A–B) fly (A, essentiality data from FlyBase; B, essentiality data from genome-wide RNAi screens in cell lines, note different scale on *x*-axis), (C–D) human (C, inferred for human genes using essentiality data for orthologs in mouse; D, essentiality data from genome-wide RNAi screens in cell lines), and (E) yeast (essentiality screens in rich medium). In fly and human, a higher fraction of multifunctional genes are essential (significance computed with the hypergeometric test). (F) Boxplots showing the number of conditions in which a gene is essential for yeast genome-wide homozygous (left) and heterozygous (right) gene deletion screens across a variety of conditions. Colored dots show the means, notches show bootstrap-generated 95% confidence intervals around the medians, boxes show quartile ranges, and whiskers extend to the most extreme data points within 1.5 times the size of the inner quartile range. Though multifunctional yeast genes are not more likely to be essential for growth in rich medium (as seen in E), they tend to be essential in a significantly larger number of conditions (Mann–Whitney U test).

For fly, we call essential all genes with a lethal phenotype (as curated by FlyBase [30]) and observe that 74% of multifunctional genes are essential, whereas only 44% of other annotated genes are essential (*p* < 2*e*-86, hypergeometric test; Fig 6A). In addition, we use data from genome-wide RNAi screens in cell lines [40] and observe that, even though only a small fraction of genes in the study overall are detected as essential, multifunctional genes have a significantly higher fraction of essential genes than other annotated genes do (3.8% and 2.9%, respectively, *p* < 0.046; Fig 6B).

For human, we call essential all genes that have a mouse ortholog with a lethal phenotype (according to MGI [41]). We find that 53% of multifunctional genes are essential, whereas only 42% of other genes are (*p* < 7*e*-16; Fig 6C). Using data from a genome-wide RNAi screen in human mammary cells [42], we also observe that multifunctional genes are essential significantly more often (*p* < 1*e*-34; Fig 6D). In a more detailed analysis using quantitative data about essentiality in 72 human cancer cell lines [43, 44], we confirm that in all 72 cell lines, multifunctional genes tend to be more essential (S3 Fig).

Gene essentiality has been found to correlate with evolutionary rate [45, 46], and we observe that multifunctional genes tend to be more evolutionarily conserved; thus, the increased evolutionary conservation of multifunctional genes could potentially explain their preferential essentiality. We confirm that whether a gene is essential is correlated with its evolutionary conservation, but observe that multifunctional genes are still significantly more essential when controlling for evolutionary conservation (see Section 1.4 in S1 Text). We note, however, that the relationship between multifunctionality and evolutionary conservation becomes much weaker when controlling for essentiality, and thus the tendency of essential genes to be more evolutionarily conserved may indeed explain the tendency of multifunctional genes to be more evolutionarily conserved (see Section 1.4 in S1 Text).

In contrast to fly and human, for yeast, when using information about essentiality for growth in rich medium, we do not observe a significant difference in essentiality: 24% of multifunctional genes and 26% of other annotated genes are essential (*p* = 0.11; Fig 6E). However, in a genome-wide screen of yeast homozygous and heterozygous deletion strains across a variety of conditions, up to 97% of yeast genes are reported as essential in at least one condition [47]. Using these data, we count the number of conditions in which each gene is detected as essential, and find that multifunctional genes are essential in a significantly larger number of conditions than are other annotated genes (*p*-values 2*e*-04 and 3*e*-03 for homozygous and heterozygous screens, respectively; Fig 6F).

### Multifunctional genes are more often involved in human disorders

**Fig 7:**
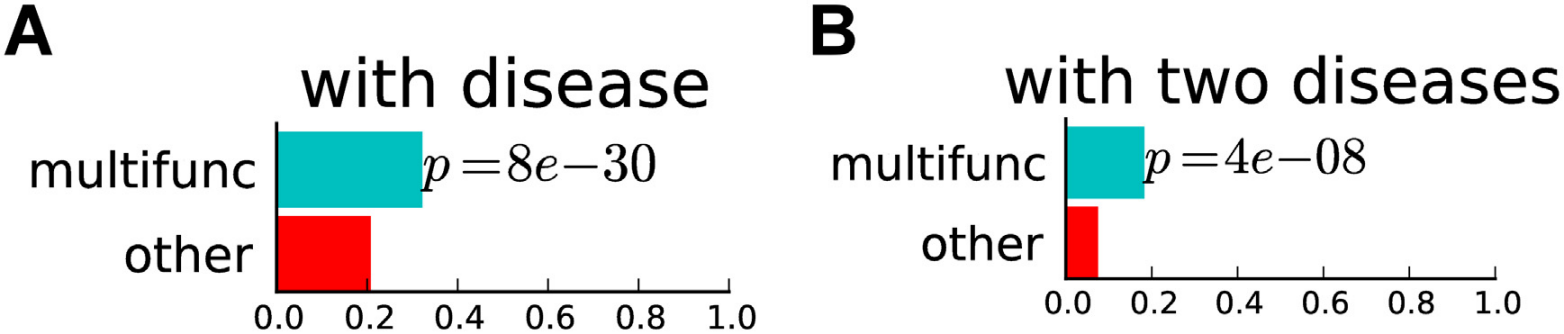
Multifunctional genes in human are associated with more diseases. (A) Barplot showing the fraction of multifunctional and other annotated human genes that are associated with a disease. (B) Barplot showing, for genes associated with disease, the fraction of multifunctional and other annotated human genes that are associated with two or more diseases. Multifunctional genes are more likely to be associated with a significantly larger number of diseases (hypergeometric test).

As multifunctional genes are more critical than other genes for the survival and normal functioning of the cell, they may potentially also be more likely to be associated with diseases. To address the relationship between gene multifunctionality and involvement in human disorders, we use the gene-disease “morbid map” from the Online Mendelian Inheritance in Man (OMIM) catalog [48], and calculate the fraction of genes with an OMIM annotation among multifunctional genes found for human. We find that 32% of all multifunctional genes are involved in at least one Mendelian disorder, whereas the fraction of other annotated genes involved in at least one Mendelian disorder is 21% (*p* < 8*e*-30, hypergeometric test; Fig 7A).

To further investigate the relationship between multifunctional genes and their involvement in human disorders, we look at genes involved in multiple distinct disorders. We map OMIM terms onto the Disease Ontology [49] and identify genes with at least one pair of disjoint OMIM terms (i.e., diseases that fall into separate branches of the Disease Ontology). We consider these genes to be involved in two or more distinct diseases. When considering genes involved in at least one disease from the Disease Ontology, we find that 18% of multifunctional genes are involved in at least two diseases, while only 8% of other such genes are involved in at least two diseases (*p* < 4*e*-8; Fig 7B).

One might expect that genes involved in more disorders, as well as multifunctional genes, are more actively studied by the research community, and that this could potentially introduce a study bias affecting our observations [12]. Using the number of PubMed publications associated with a gene as a proxy for how well studied it is, we indeed confirm that multifunctional genes are more actively studied (S4 Fig); however, even when only comparing gene sets with the same number of associated publications, we observe that the fraction of genes associated with disease is higher for multifunctional genes than for other genes (S5 Fig).

Overall, we observe that multifunctional genes are associated with diseases significantly more often than are other annotated genes.

### Multifunctional genes tend to be more central in protein interaction networks

Genes associated with multiple functions may potentially play a more central role in the global functional organization of the cell. Large-scale networks of physical protein-protein interactions provide a comprehensive view of the cellular functional landscape. In order to study how multifunctional genes are positioned in protein interaction networks, we use interaction data curated by BioGRID [39]. We use three measures of centrality: degree, betweenness centrality, and participation coefficient. Degree is the number of interactions in which a protein is involved. Betweenness centrality is the number of shortest paths passing through a node in the network, and nodes with higher betweenness are more globally central in the network. Participation coefficient shows how well a protein’s interacting partners are distributed among clusters in the network, so that proteins with low participation are mostly interacting with proteins from the same cluster, whereas proteins with high participation have their interactions spread across many clusters.

**Fig 8:**
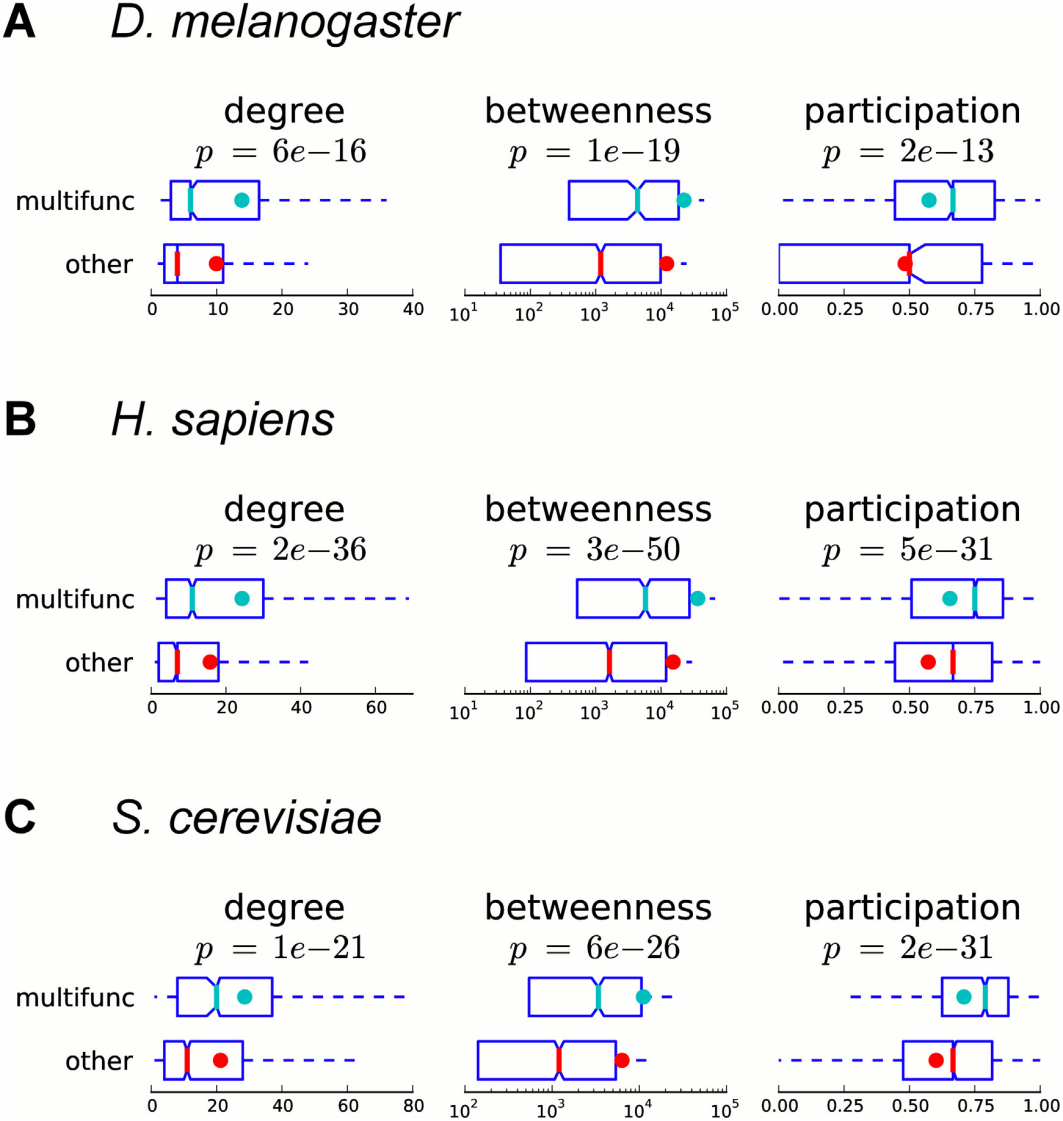
Multifunctional genes are more central in protein physical interaction networks. Boxplots of degree (number of interactions), betweenness centrality and participation coefficient of multifunctional and other annotated genes in the BioGRID protein physical interaction network are shown for (A) fly, (B) human, and (C) yeast. Colored dots show the means, notches show bootstrap-generated 95% confidence intervals around the medians, boxes show quartile ranges, and whiskers extend to the most extreme data points within 1.5 times the size of the inner quartile range. According to all three measures of centrality, multifunctional genes are significantly more central than other genes (Mann–Whitney U test).

We observe that with respect to all three considered measures, multifunctional genes are significantly more central than other genes (*p*-values from 2*e*-13 to 3*e*-50, Mann–Whitney U; Fig 8). However, not surprisingly, degree is correlated with betweenness and participation (S6 Fig), and thus the correlation between multifunctionality and degree could potentially explain the correlation with the other two more complex measures. In order to test for this, we compare multifunctional and other annotated genes with respect to their betweenness and participation when controlling for degree distribution, and still observe that multifunctional genes have significantly larger betweenness and participation (S6 Fig and S2 Table).

In order to show that our observations are not affected by potential study biases, we repeat the comparisons of degree, betweenness, and participation between multifunctional and other annotated genes in networks containing only interactions from high-throughput experiments (as reported in BioGRID [39] and HINT [50]) and observe similar results (S7 Fig). Furthermore, in order to show that potential bias in the selection of baits in these high-throughput experiments does not affect our conclusions, we compare only the number of bait-to-prey interactions reported in these high-throughput experiments. In particular, we only compare multifunctional and other genes that are baits in these experiments, and observe the same trends (S7 Fig). Overall, we conclude that multifunctional genes are more centrally positioned in protein interaction networks, and this suggests that they may play an intermodular role within interactomes.

### Same observations for multifunctionality defined with respect to molecular functions instead of biological processes

The main focus of our analysis thus far has been on multifunctional genes detected using the Biological Process ontology. However, the same procedure for detecting multifunctional genes can be applied to the Molecular Function ontology instead, thereby providing an orthogonal view of gene multifunctionality. For clarity, in this section we call the genes detected as multifunctional using the Biological Process ontology as BP-multifunctional and those detected as multifunctional using the Molecular Function ontology as MF-multifunctional.

We identify sets of MF-multifunctional genes for each organism and observe that MF-multifunctional genes have the same distinct biological properties when compared with other annotated genes as has been reported in the previous sections for BP-multifunctional genes (although some *p*-values for yeast are above our significance threshold of 5%; see S8, S9 and S10 Figs).

In order to see if the involvement of a gene in multiple biological processes (BP-multifunctional) can be explained by multiple functions of the gene at the molecular level (MF-multifunctional), we directly compare the two sets of multifunctional genes derived from the two ontologies. We observe that 12% to 35% of BP-multifunctional genes are also MF-multifunctional, which constitutes a significant overlap (*p* < 6*e*-18; S3 Table), while the remainder may potentially be explained by other modes of gene multifunctionality. In contrast, a gene involved in multiple molecular functions might be expected to have these molecular functions while performing different biological processes, and indeed most MF-multifunctional genes are also BP-multifunctional (56% to 78%; S4 Table). These results are consistent with previous observations made using a simpler multifunctionality definition counting leaf GO terms associated with each protein [15]. Note, however, that the total number of MF annotations is lower than the total number of BP annotations (S3 and S4 Tables), and thus the total number of genes identified as MF-multifunctional is lower than the total number of genes identified as BP-multifunctional (S4 Table).

## Discussion

Most proteins are—at least to some extent—multifunctional. Even within this context, previous experimental studies have identified proteins that perform remarkably different molecular functions [2–6], or that affect several distinct biological processes [7, 8, 10]. These findings suggest the existence of a subset of genes that are endowed with a particularly high degree of functional plasticity. There is increasing evidence that the phenomenon of gene multifunctionality is actually very common; thus, studying multifunctionality at a systems level can help elucidate the functional organization of the cell. In this paper, we introduce a computational approach to systematically identify multifunctional genes using existing functional annotations, and show that multifunctional genes are characterized by distinct properties as compared to other genes. To the best of our knowledge, our work represents the largest-scale characterization of gene multifunctionality to date, with whole-genome analysis across several organisms.

As compared to other studies, our approach specifically addresses some previous weaknesses in handling GO functional annotations. In several previous publications, a simple count of the number of GO terms annotating a gene was used as a proxy for gene multifunctionality [11, 12, 19]. However, idiosyncrasies of GO may result in similar functions or processes being categorized in distinct places in the ontology. In our approach to identify multifunctional genes, we explicitly select semantically distinct terms that co-occur less frequently than expected by chance. Special care is also taken in gauging the effects of study bias, particularly in the case of interaction network properties and disease genes; that is, multifunctional genes may appear more often in the results of various experiments and thus be more actively studied by researchers, and this could potentially introduce a study bias in our analysis. In order to avoid this, we mostly analyze high-throughput and whole-genome data sets. When looking at associations of multifunctional genes with manually curated data (e.g., association with diseases), which could potentially suffer from study bias, we directly correct for this bias. Further, we carry out inter-species comparisons, and observe similar trends across three different organisms, thereby minimizing the effects of organism-specific annotation biases.

A remaining challenge in characterizing multifunctional genes at the genome-wide level is that current knowledge about gene function is far from complete; thus, new experimental information about the function of some genes could result in their reclassification as multifunctional. In the limit, we may expect that nearly all genes are, to varying degrees, multifunctional. Nevertheless, the robustness of our results—both across a diverse set of organisms with distinct functional annotations and biases as well as within a single organism when explicitly controlling for study bias—suggests that we have identified specific biological features that are associated with the degree of functional plasticity of a gene.

We find that gene multifunctionality is associated with several distinct properties that have important functional consequences. In the protein interactome, multifunctional proteins have a tendency to occupy more central and intermodular regions, even after controlling for potential study bias; this suggests that multifunctional proteins connect distinct and more specialized parts of the interactome, and are critical for information flow within the cell. Consistent with their important role within the cell, we also observe that multifunctional genes are more likely to be essential and are more often found to be associated with diseases. At the expression level, multifunctional genes are more broadly expressed across different conditions or cell types than are other genes. It is therefore possible that only subsets of functions are performed by multifunctional proteins under specific conditions or in particular cell types. We also observe that the expression of multifunctional genes appears to be finely regulated, as it involves a larger number of transcription factors than expected. At the molecular level, we find that multifunctional proteins have a larger number of unique domains as compared to other proteins; this is consistent with the wider spectrum of functions that they carry out. However, consistent with previous reports [25], we also find that multifunctional proteins have a higher degree of structural disorder. Determining which of these properties or combinations of properties represent the main mechanism underlying the functional plasticity of a gene is of great interest. It is also possible to speculate that multifunctionality may be achieved via class-specific mechanisms where certain mechanisms may be at play only for a given class of genes.

As part of our analysis, we perform a cross-genomic analysis of gene multifunctionality. We find that multifunctional genes are more evolutionarily conserved than other genes; this may be due to their being under stronger evolutionary pressure as they perform multiple functions, with different functions potentially performed in different conditions. Further, orthologous genes tend to share their propensity for multifunctionality; this suggests that the multifunctionality of many genes may have an early evolutionary origin. It also further supports the validity of our method to detect multifunctional genes, as they are uncovered in each organism independently.

Our method to detect genes annotated with distinct functional terms can be applied to any of the vocabularies in GO, and this allows us to look at the phenomenon of gene multifunctionality from different perspectives. We observe, not surprisingly, that most genes identified as being involved in multiple molecular functions are also identified as participating in multiple biological processes. However, we detect many genes involved in multiple biological processes for which there is no evidence of association with multiple molecular functions. While this may be partly due to the fewer number of molecular function annotations, it also suggests that these genes may perform the same molecular function while carrying out different biological processes, depending upon a spatio-temporal context. Being able to tease apart the conditions under which a specific function is performed by a gene is an important avenue for future research in functional genomics, and could even lead to the development of a context-specific GO vocabulary. In this ontology, the terms used to annotate genes could be qualified with other terms specifying the cell type, the developmental stage, or the stage in the cell-cycle in which a given function is most likely to be carried out by a gene.

In conclusion, a comprehensive understanding of gene and protein function has been a major goal of computational biology since the emergence of the field. In this work, we develop a computational method for genome-wide detection of multifunctional genes using existing functional annotations. We make a number of novel observations about gene multifunctionality across several organisms, as well as confirm some previous findings (including many cases where only anecdotal evidence existed). Overall, our work contributes to a better systematic understanding of the functional landscape of the proteome, and can be the basis for future work in this direction as more specific and detailed functional genomics data become available.

## Materials and Methods

### Multifunctional genes

Gene Ontology (GO) [22] terms and gene association data for each organism were downloaded from http://www.geneontology.org/ on July 12, 2013. For the main analysis reported in the paper, we include all functional associations with evidence codes EXP (“Inferred from Experiment”), IDA (“Inferred from Direct Assay”), IMP (“Inferred from Mutant Phenotype”), IGI (“Inferred from Genetic Interaction”), IEP (“Inferred from Expression Pattern”), ISS (“Inferred from Sequence or structural Similarity”), ISO (“Inferred from Sequence Orthology”), ISA (“Inferred from Sequence Alignment”), ISM (“Inferred from Sequence Model”), IGC (“Inferred from Genomic Context”), IBA (“Inferred from Biological aspect of Ancestor”), IC (“Inferred by Curator”), TAS (“Traceable Author Statement”), and NAS (“Non-traceable Author Statement”). We exclude all annotations with the qualifier NOT. We also perform additional analyses restricting ourselves to GO annotations with evidence codes EXP, IDA, IMP, IEP, IC, and TAS; these results are consistent with those reported in the main body of the paper (see S11, S12, S13 Figs and S6 File). For all GO analysis, we use code from the project goatools (https://github.com/tanghaibao/goatools).

We call multifunctional every gene that is annotated with at least “two sufficiently distinct functional terms of comparable specificity,” as explained next. First, to define terms of about equal specificity, we start with the notion of informative terms used previously in the literature [51–54], which selects for a given *N* all terms that annotate ≥ *N* genes, but whose descendant terms annotate < *N* genes. However, we observe that a very general term annotating many genes may have all descendant terms annotating only small numbers of genes, even if it annotates many more than *N* genes. For example, a fly term imaginal disc-derived wing morphogenesis (GO:0007476) annotates 508 genes, but its descendant terms annotate no more than 82 genes each (248 genes in total), and it may be undesirable to call this term informative for *N* ≈ 100, as it is actually a much more general term than terms that annotate approximately 100 genes. To overcome this problem, we select the set *T*_*N*_ of all terms which annotate ≥ *N* genes, but < 2*N* genes, and whose every descendant term annotates < *N* genes (this includes terms with no descendants). Next, from all genes annotated by terms from *T_N_* we extract the genes annotated with at least two such terms. In order to consider annotations by distinct terms only, from the collection of all pairs of selected terms {(*t*_1_, *t*_2_): *t*_1_, *t*_2_ ∈ *T_N_*}, we further select pairs of terms that are sufficiently distinct. First, we filter out pairs of terms that have a common descendant term, as this may be an indication of similarity between the terms. We also remove all pairs of terms that have pairwise semantic similarity larger than zero [55]; though alternate thresholds of semantic similarity could be used, here we select only pairs of terms whose least common ancestor is the root of the ontology. Finally, terms annotating similar sets of genes may correspond to similar functions, so we filter out all pairs of terms that annotate significantly overlapping sets of genes (hypergeometric test, *p* < 0.1). A gene co-annotated by some pair of selected terms from *T_N_* passing these filters is called multifunctional. In order to focus on more specific biological process terms and avoid considering less informative (i.e., more general) terms annotating a lot of genes, we require that *N* is not greater than a certain threshold *M*; we choose *M* = 120 for the analysis in the main text. The final set of multifunctional genes is given by the union of all sets obtained for different *N*, where *N* ranges from 10 up to *M*, with an increment of 10. We compare multifunctional genes with all other genes that are annotated with any selected term from *T_N_* for *N* between 10 and *M* (with an increment of 10). We show that, for all our results, the same trends are observed when varying the parameter *M* (S4 File), the *p*-value threshold in co-annotation filter (S5 File), and when restricting the analysis to a subset of the most reliable GO annotations (S11, S12, S13 Figs and S6 File).

### Data sources

#### Physicochemical properties of genes

The proteomes of *D. melanogaster*, *H. sapiens*, and *S. cerevisiae* were downloaded from UniProt (September 2013 release). For each protein encoded by a gene, we compute its amino acid sequence length, number of domains and average disorder as follows. For fly and human, we consider the longest protein isoform encoded by a gene (for yeast, there is only one isoform per gene in the database). Domain information was obtained from Pfam 27.0 [56], using the annotations contained in the swisspfam file. For each protein sequence, the number of unique protein domain families found within it is computed; that is, multiple instances of the same domain family are ignored. Predictions of disordered residues are carried out using the IUPred program [26, 27], with default parameters. The average disorder of the protein is then computed as the fraction of residues with a IUPred score above 0.5, as suggested by the authors.

#### Expression

*D. melanogaster*: FlyAtlas project data [28] was downloaded from GEO [57] (accession number GSE7763). A gene is considered present in a tissue or condition if it is detected as present in all replicates, as reported in the dataset. We also use RNA-seq data from modENCODE [29] as processed by FlyBase [30, 58]. A gene with non-zero RPKM in a tissue is considered present in this tissue. *H. sapiens*: Expression data for human was downloaded using Ensembl BioMart, release 73 [31], using data sources “GNF/Atlas organism part” for GNF Atlas [59] and “Anatomical System (egenetics)” for eGenetics [60].

#### Evolutionary conservation

Evolutionary conservation scores from phastCons [32] were downloaded from the UCSC genome browser website on December 10, 2013, for *D. melanogaster*, *H. sapiens*, and *S. cerevisiae* [61]. Conservation scores are averaged over nucleotides of exons for each isoform and then averaged over isoforms.

#### Regulatory interactions

Regulatory transcription factor–gene interactions were obtained from DroID [35], version v2013_07 for *D. melanogaster*; from ENCODE [36, 62] for *H. sapiens*; and from YeastMine [63] (downloaded November 24, 2013, high-throughput interactions attributed to [37] or [38]) for *S. cerevisiae*.

#### Genetic interactions

Genetic interactions for fly were obtained from FlyBase [30], version v2013_07, and for yeast from BioGRID [39], version 3.2.102. For yeast, positive (evidence codes Positive Genetic, Synthetic Rescue) and negative (evidence codes Negative Genetic, Synthetic Growth Defect, Synthetic Lethality) genetic interactions are also considered separately.

#### Essentiality

Phenotype data were obtained for fly (FlyBase [30], version v2013_07), mouse (MGI [41], downloaded October 3, 2013), and yeast (from YeastMine [63], downloaded September 26, 2013). Essential genes are defined as genes with a “lethal” phenotype for fly and with an “inviable” phenotype for yeast. For human, genes are deemed essential if their mouse orthologs (as reported by MGI) have associated with them any phenotype containing “lethal” in its name. When applying a hypergeometric test for enrichment of essential genes in multifunctional genes, the set of all genes with any reported phenotype is used as a background. In addition, sets of essential genes detected in genome-wide RNAi screens in cell lines were obtained from OGEE [64] for fly [40] and human [42]. A more detailed analysis reporting a score of essentiality for each gene in a genome-wide screen in each of 72 tested human cancer cell lines was obtained from COLT-Cancer [43, 44, 65]. For yeast, we also use data from genome-wide heterozygous and homozygous gene deletion screens across multiple conditions [47], and for each gene count the number of conditions for the corresponding deletion strain with *p*-value below 0.01 [66].

#### Disease data

We used BioMart [67] to obtain gene-disease associations from the Online Mendelian Inheritance in Man (OMIM) catalog [48]. Out of the 9664 human genes annotated with at least one GO term used in the definition of multifunctionality (see Subsection **Multifunctional genes**), 2299 had at least one OMIM association. To further probe the similarities between diseases involving the same genes, we used the Disease Ontology [49], a knowledgebase of human disorders that are hierarchically organized in a directed acyclic graph. We mapped OMIM terms to Disease Ontology terms using the OBO file available at http://disease-ontology.org/downloads. Out of 2299 genes with an OMIM association, 1148 have at least one Disease Ontology term. We focus on genes with at least one Disease Ontology term, and extract from them all genes that have at least two Disease Ontology terms with only the root node in common; this results in 135 genes, which we consider as genes associated with at least two distinct diseases.

#### Physical protein-protein interactions

Physical protein-protein interactions were obtained from BioGRID [39], version 3.2.102. We iteratively remove proteins with more than 200 interactions, as proteins may have large numbers of interactions due to experimental artifacts (i.e., in each iteration, the protein with the highest number of interactions is removed along with its interactions). To extract high-throughput interactions, we consider only interactions indicated as high-throughput in the database and only from publications contributing interaction data with at least 100 baits. For human and yeast, we also consider high-throughput interaction datasets from HINT [50].

#### PubMed publications

The number of PubMed publication IDs associated with each gene was downloaded from NCBI at http://www.ncbi.nlm.nih.gov/gene on September 18, 2013.

#### External databases

Lists of multitasking and moonlighting proteins were obtained from the MultitaskProtDB (http://wallace.uab.es/multitask/) [23] and MoonProt (http://www.moonlightingproteins.org/) [24] databases on April 7, 2015. Both databases curate the lists of proteins experimentally verified to have multiple biological functions.

### Comparison across orthologs

Protein ortholog information was obtained from version 4 of the Princeton Protein Orthology Database (P-POD) [34, 68]. Two proteins from different organisms are considered orthologous if they belong to the same family, as detected by P-POD using either OrthoMCL or MultiParanoid. For each pair of organisms, we compute how many orthologous pairs of multifunctional genes are found where one gene in a pair is from one organism and the other gene in the pair is from the other organism. To assess significance, we repeat the computation 1000 times with randomization. In each random trial, we permute the labels of multifunctional and other annotated genes within each organism, while considering only genes involved in orthologous relationships. The orthology relationship between genes of different organisms is preserved. Then we compute the average and standard deviation of the counts in random trials along with an empirical *p*-value of the real count with respect to the randomized counts.

### Network analysis

The **degree** of a vertex is the number of interactions that the corresponding protein has in the network. The **betweenness centrality** of a vertex *v* is the number of shortest paths between all pairs of vertices in the network that pass through *v*, with the shortest paths between two vertices *s* and *t* weighed inversely to the total number of distinct shortest paths between them. The **participation coefficient** [69, 70] of a vertex with respect to a set of clusters in a network is defined as $P=1-\sum_{i}\;{\left(\frac{k_{i}}{k}\right)}^{2}$, where the summation is over all clusters, *k* is the vertex degree, and *k_i_* is the number of edges going from the vertex to vertices from the cluster *i*. The rationale is to have *P* = 0 if all edges from the vertex go to a single cluster, and to have *p* closer to 1 if edges from the vertex are more uniformly distributed over clusters. To find clusters in the network, we use the SPICi clustering algorithm [71] with parameters optimized with a simple exhaustive search procedure to approximately maximize Newman’s modularity [72], as described earlier [73]. For network analysis, we use the python interface to the igraph library, version 0.6.5 (http://igraph.sourceforge.net/).

## Supporting Information

S1 Text Supporting Results and Methods. (PDF)

S1 Fig Effect of varying parameters in the definition of multifunctional genes. (A-C) Terms chosen at different specificity levels. The number of Biological Process (BP) Gene Ontology (GO) terms chosen is shown for each specificity threshold from 10 to 200 (increment of 10) for (A) fly, (B) human, (C) yeast. (D-F) Genes annotated with terms chosen at different specificity levels. For each *M* from 10 to 200 (increment of 10), the cumulative number of genes annotated with terms chosen for specificity thresholds *N* ≤ *M* is shown for (D) fly, (E) human, (F) yeast. Horizontal line shows the total number of genes annotated with any BP term. (G-I) Fraction of multifunctional genes in all annotated genes. For each specificity threshold, the fraction of the cumulative number of multifunctional genes to the total number of all genes annotated with terms chosen at this threshold is shown for (G) fly, (H) human, (I) yeast. See Materials and Methods for details.
(PNG)

S2 Fig Multifunctional genes have more isoforms in fly and human. Boxplots of the number of isoforms per gene for multifunctional and other annotated genes in (A) fly and (B) human. Multifunctional genes have significantly larger number of isoforms (Mann–Whitney U). (C) Boxplots of the number of conditions in which multifunctional and other annotated genes in fly are expressed, for genes with one isoform only (which constitute 49% of multifunctional genes and 59% of other annotated genes). (D) Boxplots of the number of organism parts in which multifunctional and other annotated genes in human are expressed, for genes with 2 to 5 isoforms (17% of multifunctional and 18% of other genes have 2 isoforms, 14% of multifunctional and 14% of other genes have 3 isoforms, 10% of multifunctional and 11% of other genes have 4 isoforms, 9% of multifunctional and 8% of other genes have 5 isoforms). See Fig 3 for comparison across all genes.
(PNG)

S3 Fig Multifunctional genes are more essential in human cancer cell lines. For each of 72 human cancer cell lines (*x*-axis) in the COLT-Cancer database [43, 44], the median GARP score of essentiality, as reported in the database, is shown for multifunctional (cyan) and all other annotated (red) genes on the *y*-axis; lower GARP scores depict higher essentiality. Multifunctional genes tend to be more essential than other annotated genes in all 72 cell lines.
(PDF)

S4 Fig Multifunctional genes have been more studied than other genes. Boxplots of the number of PubMed publications associated with multifunctional and other annotated genes are shown for (A) fly, (B) human, and (C) yeast. Multifunctional genes are associated with a significantly larger number of publications (Mann–Whitney U test).
(PNG)

S5 Fig Comparison of the association of multifunctional and other annotated human genes with diseases, when controlling for study bias. Fractions of multifunctional (cyan) and other annotated (red) genes associated with diseases are shown (same as in Fig 7), as well as the estimated fractions in other genes after controlling for study bias (olive, with the boxes giving the 95% confidence intervals). The estimation is from 1000 independent random samples from the set of other annotated genes, where the samples have the same distribution of the number of associated publications as multifunctional genes. Multifunctional genes are associated with significantly larger number of diseases even after controlling for study bias (empirical *p*-values shown). See **Methods** in S1 Text for details.
(PDF)

S6 Fig Comparison of centrality in protein-protein physical interaction networks of multifunctional and other annotated genes, when controlling for degree distribution. (A) Barplots of the Spearman correlations between degree and betweenness centrality and participation coefficient, as measured for fly, human and yeast physical protein-protein interaction networks. Degree is highly correlated with both measures. (B–D) Comparison of betweenness and participation of multifunctional and other annotated genes, while controlling for degree. Boxplots show the distribution of betweenness or participation for multifunctional and other annotated genes for (B) fly, (C) human, (D) yeast (same as in Fig 8). In magenta are the distributions of the same measures for random samples from the set of other annotated genes, where the samples have the same degree distribution as multifunctional genes. Vertical magenta lines show the estimated medians, boxes show the 95% confidence intervals around the medians, and horizontal lines show the 25%–75% quantile ranges. After controlling for degree, the betweenness and participation of multifunctional genes are significantly higher than for other annotated genes (empirical *p*-value computed for comparing medians). See **Methods** in S1 Text for details.
(PNG)

S7 Fig Centrality of multifunctional genes in high-throughput protein physical interaction networks. Boxplots with three measures of centrality—degree (number of interactions), betweenness centrality, and participation coefficient—in high-throughput protein interaction networks. Comparisons of multifunctional and other annotated genes are shown for (A) fly (BioGRID), (B) human (HINT), (C) human (BioGRID), (D) yeast (HINT), (E) yeast (BioGRID). Multifunctional genes are significantly more central than other annotated genes (Mann–Whitney U test) in high-throughput networks that are not prone to bias towards more studied genes. For an even stricter comparison, the degree of bait genes—i.e., the number of interactions from bait to prey genes in these high-throughput experiments—is compared between multifunctional and all other annotated genes, and the trend is confirmed in all networks (A–E).
(PNG)

S8 Fig Analysis of multifunctional genes in *D. melanogaster* obtained using the Molecular Function ontology. Comparison of multifunctional and other annotated genes obtained from the Molecular Function Gene Ontology using our method (see Fig 1 and Materials and Methods). (A) Physicochemical properties (compare with Fig 2A). (B) Expression (compare with Fig 3A). (C) Evolutionary conservation (compare with Fig 4A). (D) Regulatory and genetic interactions (compare with Fig 5A). (E) Essentiality (FlyBase curated; compare with Fig 6A). (F) Centrality in protein-protein interaction networks (compare with Fig 8A).
(PNG)

S9 Fig Analysis of multifunctional genes in *H. sapiens* obtained using the Molecular Function ontology. Comparison of multifunctional and other annotated genes obtained from the Molecular Function Gene Ontology using our method (see Fig 1 and Materials and Methods). (A) Physicochemical properties (compare with Fig 2B). (B) Expression (compare with Fig 3B). (C) Evolutionary conservation (compare with Fig 4B). (D) Regulatory interactions (compare with Fig 5B). (E) Essentiality (mouse orthologs; compare with Fig 6C). (F) Association with diseases (compare with Fig 7). (G) Centrality in protein-protein interaction networks (compare with Fig 8B).
(PNG)

S10 Fig Analysis of multifunctional genes in *S. cerevisiae* obtained using the Molecular Function ontology. Comparison of multifunctional and other annotated genes obtained from the Molecular Function Gene Ontology using our method (see Fig 1 and Materials and Methods). (A) Physicochemical properties (compare with Fig 2C). (B) Evolutionary conservation (compare with Fig 4C). (C) Regulatory and genetic interactions (compare with Fig 5C). (D) Essentiality (compare with Fig 6E and 6F). (E) Centrality in protein-protein interaction networks (compare with Fig 8C).
(PNG)

S11 Fig Analysis of multifunctional genes in *D. melanogaster* obtained using the most reliable GO evidence codes. Comparison of multifunctional and other annotated genes obtained from the most reliable GO BP annotations (with evidence codes EXP, IDA, IMP, IEP, IC, TAS) using our method (see Fig 1 and Materials and Methods). (A) Physicochemical properties (compare with Fig 2A). (B) Expression (compare with Fig 3A). (C) Evolutionary conservation (compare with Fig 4A). (D) Regulatory and genetic interactions (compare with Fig 5A). (E) Essentiality (FlyBase curated; compare with Fig 6A). (F) Centrality in protein-protein interaction networks (compare with Fig 8A).
(PNG)

S12 Fig Analysis of multifunctional genes in *H. sapiens* obtained using the most reliable GO evidence codes. Comparison of multifunctional and other annotated genes obtained from the most reliable GO BP annotations (with evidence codes EXP, IDA, IMP, IEP, IC, TAS) using our method (see Fig 1 and Materials and Methods). (A) Physicochemical properties (compare with Fig 2B). (B) Expression (compare with Fig 3B). (C) Evolutionary conservation (compare with Fig 4B). (D) Regulatory interactions (compare with Fig 5B). (E) Essentiality (mouse orthologs; compare with Fig 6C). (F) Association with diseases (compare with Fig 7). (G) Centrality in protein-protein interaction networks (compare with Fig 8B).
(PNG)

S13 Fig Analysis of multifunctional genes in *S. cerevisiae* obtained using the most reliable GO evidence codes. Comparison of multifunctional and other annotated genes obtained from the most reliable GO BP annotations (with evidence codes EXP, IDA, IMP, IEP, IC, TAS) using our method (see Fig 1 and Materials and Methods). (A) Physicochemical properties (compare with Fig 2C). (B) Evolutionary conservation (compare with Fig 4C). (C) Regulatory and genetic interactions (compare with Fig 5C). (D) Essentiality (compare with Fig 6E and 6F). (E) Centrality in protein-protein interaction networks (compare with Fig 8C).
(PNG)

S14 Fig Evolutionary conservation analysis of multifunctional genes when restricting GO annotations. Boxplots of evolutionary conservation (estimated by phastCons [32] for each nucleotide, averaged over the nucleotides of each gene) of multifunctional and other annotated genes are shown for (A) fly, (B) human, and (C) yeast. When detecting multifunctional genes, from the set of annotations used in the analysis in the main text (see Materials and Methods), those with evidence codes ISS, ISA, ISO, ISM are removed. Colored dots show means, notches show bootstrap-generated 95% confidence intervals around the medians, boxes show quartile ranges, and whiskers extend to the most extreme data points within 1.5 times the size of the inner quartile range. Multifunctional genes are significantly more evolutionary conserved than other genes (Mann–Whitney U test). Compare with Fig 4.
(PNG)

S1 Table Genes with more isoforms tend to be detected as more broadly expressed. Spearman correlations (with *p*-values) between the number of isoforms of a gene and the number of tissues or organism parts in which the gene is expressed, according to genome-wide assays in fly and human (see main text and Materials and Methods).
(PDF)

S2 Table Multifunctionality and centrality in protein-protein physical interaction networks. In the first part of the table, we show Spearman correlations (with *p*-values) between whether a gene is multifunctional (1 or 0 depending on whether it is found to be multifunctional or not) and its degree, betweenness, and participation in protein-protein interaction networks. All correlations are positive and significant (compare with Fig 8). In the second part of the table, we show partial Spearman correlations between gene multifunctionality and betweenness and participation, when controlling for degree. All partial correlations are small but positive, and are statistically significant (compare with S6 Fig).
(PDF)

S3 Table Comparison of BP-multifunctional to MF-multifunctional genes. Analysis of multifunctional genes derived from the Biological Process ontology (BP-multifunctional) using the specificity parameter upper bound 120 (the same as used in the main analysis of the paper; see Figs 2, 3, 4, 5, 6, 7, 8), when compared with multifunctional genes derived from the Molecular Function ontology (MF-multifunctional) using the specificity parameter upper bounds 120 (a more specific cut-off) and 500 (a more general cut-off allowing more genes to be detected as MF-multifunctional). For each organism, shown is the number of BP-multifunctional genes (see Table 1); the number of them annotated with specific terms from MF; the number and percent of such genes that are detected as MF-multifunctional; and the *p*-value from the hypergeometric test corresponding to the significance of this intersection. A significant fraction of BP-multifunctional genes are also MF-multifunctional.
(PDF)

S4 Table Comparison of MF-multifunctional to BP-multifunctional genes. Analysis of multifunctional genes derived from the Molecular Function ontology (MF-multifunctional) using the specificity parameter upper bound 120 (used in the analysis shown in S8, S9, S10 Figs) when compared with multifunctional genes derived from the Biological Process ontology (BP-multifunctional) using the specificity parameter upper bounds 120 (a more specific cut-off) and 500 (a more general cut-off allowing more genes to be detected as BP-multifunctional). For each organism, shown is the number of MF-multifunctional genes; the number of them annotated with specific terms from BP; the number and percent of such genes that are detected as BP-multifunctional; and the *p*-value from the hypergeometric test corresponding to the significance of intersection. Most MF-multifunctional genes are also BP-multifunctional.
(PDF)

S1 File Information about multifunctional genes and other gene characteristics in *D. melanogaster*. (TXT)

S2 File Information about multifunctional genes and other gene characteristics in *H. sapiens*. (TXT)

S3 File Information about multifunctional genes and other gene characteristics in *S. cerevisiae*. (TXT)

S4 File Results of comparing multifunctional and other annotated genes for different values of *M* (see Materials and Methods). (TXT)

S5 File Results of comparing multifunctional and other annotated genes for different values of the co-annotation *p*-value threshold (see Materials and Methods). (TXT)

S6 File Results of comparing multifunctional and other annotated genes for a subset of the most reliable GO annotations (see Materials and Methods). (TXT)
