# Supplementary material for: Genome-Wide Detection and Analysis of Multifunctional Genes
Source: PLoS Comput Biol. 2015 Oct 5;11(10):e1004467. doi: 10.1371/journal.pcbi.1004467 (PMC4593560; doi:10.1371/journal.pcbi.1004467)
Supplement: S1 Text — (PDF) [file pcbi.1004467.s001.pdf]

Supporting Text S1 for
Genome-wide Detection and Analysis of Multifunctional Genes

Yuri Pritykin   Dario Ghersi*   Mona Singh†

April 30, 2015

1 Results

1.1 Validation of the method via external databases

Validation of any method of genome-wide detection of multifunctional genes is hard, since there is no ground truth available on a genome-wide scale. Nevertheless, we considered two resources that collected experimentally validated and literature curated sets of multitasking and moonlighting proteins—databases MultitaskProtDB and MoonProt, both with a very small number of genes.

MultitaskDB contained 6 genes for fly, 84 genes for human, and 18 genes for yeast. Most of these genes were annotated by one of the terms used to detect multifunctional genes by our method: 5 for fly, 77 for human, and 18 for yeast. A significantly large fraction of these genes were actually detected as multifunctional by our method: 3 (60%; $p < 0.01$, hypergeometric test) for fly, 39 (51%; $p < 1e{-}6$) for human, and 10 (56%; $p < 8e{-}5$) for yeast.

MoonProt contained 4 genes for fly, 48 genes for human, and 27 genes for yeast. Most of these genes were annotated by one of the terms used to detect multifunctional genes by our method: 4 for fly, 44 for human, and 26 for yeast. A significantly large fraction of these genes were actually detected as multifunctional by our method: 3 (75%; $p < 3e{-}3$) for fly, 22 (50%; $p < 2e{-}4$) for human, and 13 (50%; $p < 6e{-}5$) for yeast.

Therefore we conclude that our method is successful at detecting a significant fraction of experimentally verified multifunctional genes.

1.2 Length and number of domains in multifunctional and other annotated proteins

We observe that proteins encoded by multifunctional genes are significantly longer than proteins encoded by other annotated genes (see main text and Fig. 2). Equivalently, whether a gene is multifunctional is positively correlated with the length of a protein: Spearman's $\rho = 0.17$ ($p < 6e{-}40$) for *D. melanogaster*, $\rho = 0.06$ ($p < 2e{-}9$) for *H. sapiens*, and $\rho = 0.10$ ($p < 1e{-}11$) for *S. cerevisiae*. Also, proteins encoded by multifunctional genes have a significantly larger number of unique domains than proteins encoded by other annotated genes (see main text and

*Co-corresponding author. E-mail: dghersi@unomaha.edu
†E-mail: mona@cs.princeton.edu

Fig. 2). Equivalently, multifunctionality has a significant positive Spearman correlation with the number of unique protein domains: $\rho = 0.07$ ($p < 1e{-}7$) for *D. melanogaster*, $\rho = 0.07$ ($p < 4e{-}11$) for *H. sapiens*, and $\rho = 0.06$ ($p < 1e{-}4$) for *S. cerevisiae*.

However, one may expect that longer proteins have more domains, so the difference in length could explain the difference in the number of domains between multifunctional and other annotated genes. Indeed, we observe strong significant positive Spearman correlation between protein length and the number of unique domains in all three organisms: $\rho = 0.52$ for *D. melanogaster*, $\rho = 0.65$ for *H. sapiens*, and $\rho = 0.61$ for *S. cerevisiae*. We compute the partial Spearman correlation between multifunctionality and the number of domains with a correction for protein length and observe a significant (though small) value only for human: $\rho = 0.04$ ($p < 2e{-}4$). Therefore the difference in length between multifunctional and other annotated proteins may indeed explain the significant difference in the number of domains, or the difference in the number of domains between multifunctional and other annotated proteins may indeed explain significant difference in length. Further investigation is required.

1.3 Evolutionary analysis of multifunctionality for selected GO annotations

Gene ontology annotations are in part defined by transferring information between organisms via sequence similarity, and the evolutionary trends observed between and within organisms (see main text and Fig. 4) may potentially be explained in part by this annotation transfer. In order to test this, we repeat the analysis using a subset of GO annotations where, in addition to filtering described in **Materials and methods** in the main text, we further remove from consideration all annotations whose only evidence is marked with evidence codes "Inferred from Sequence or Structural Similarity" (ISS), "Inferred from Sequence Alignment" (ISA), "Inferred from Sequence Orthology" (ISO), or "Inferred from Sequence Model" (ISM). Using our algorithm on these filtered annotations (as described in **Materials and methods** in the main text) results in 1516 genes detected as multifunctional in fly, out of 5209 genes annotated with the terms used in the algorithm; 2276 genes detected as multifunctional in human, out of 9304 genes annotated with the terms used in the algorithm; and 864 genes detected as multifunctional in yeast, out of 4514 annotated with the terms used in the algorithm (compare with Table 1 in the main text).

Using this subset of GO annotations, we still observe the same trends as presented in the main text. Evolutionary conservation for multifunctional genes is higher than for other annotated genes (though the p-value for yeast is above our significance threshold of 5%): 0.68 ± 0.15 vs. 0.64 ± 0.18 ($p < 9e{-}10$) for fly, 0.55 ± 0.17 vs. 0.52 ± 0.18 ($p < 7e{-}9$) for human, 0.77 ± 0.16 vs. 0.75 ± 0.18 ($p = 0.08$) for yeast (mean $\pm$ standard deviation and p-value from Mann–Whitney U test are shown for multifunctional and other annotated genes, respectively, in each organism).

We also observe 1615 pairs of orthologous multifunctional genes between fly and human (while 818.9 ± 86.6 are expected by chance, actual value 2.0 times higher; $p < 1e{-}3$), 397 pairs of orthologous multifunctional genes between fly and yeast (179.6 ± 21.0 expected by chance, actual number 2.2 times higher; empirical $p < 1e{-}3$), 542 pairs of orthologous multifunctional genes between human and yeast (253.5 ± 31.0 expected by chance, actual number 2.1 times higher; empirical $p < 1e{-}3$).

These results indicate that our observations on evolutionary aspects of multifunctionality presented in the main text are not explained by dependency of GO annotation assignment on

gene orthology.

1.4 Joint analysis of multifunctionality, essentiality, and evolutionary conservation

We confirm previously reported finding that gene essentiality is correlated with evolutionary conservation of genes: Spearman correlation between essentiality (1 for a gene if essential and 0 otherwise) and evolutionary conservation $\rho = 0.26$ ($p < 2.2e{-16}$) for fly (essentiality as reported in FlyBase), $\rho = 0.27$ ($p < 2.2e{-16}$) for human (essentiality obtained from orthology with mice). As we report in the main text, we also observe that multifunctional genes are more often essential. Equivalently, whether a gene is multifunctional is positively correlated with its essentiality: Spearman's $\rho = 0.27$ ($p < 2.2e{-16}$) for fly and $\rho = 0.11$ ($p < 8e{-16}$) for human. We also report in the main text that multifunctional genes are significantly more evolutionarily conserved, or equivalently, whether a gene is multifunctional is positively correlated with its evolutionary conservation: Spearman's $\rho = 0.09$ ($p < 9e{-13}$) for fly, $\rho = 0.06$ ($p < 1e{-9}$) for human. Potentially the latter may be the only explanation for the correlation of multifunctionality and essentiality. However, we compute partial Spearman correlation of multifunctionality and essentiality when correcting for evolutionary conservation and still observe significantly positive values: $\rho = 0.26$ ($p < 1e{-79}$) for fly, $\rho = 0.10$ ($p < 1e{-9}$) for human. Therefore we conclude that correlation of multifunctionality and evolutionary conservation cannot fully explain significant positive correlation between multifunctionality and essentiality. Furthermore, we also compute partial Spearman correlation of multifunctionality and evolutionary conservation when correcting for essentiality and also observe signifiantly positive values: $\rho = 0.05$ ($p < 4e{-4}$) for fly, $\rho = 0.03$ ($p < 0.02$) for human. However, weaker correlations in this case indicate that the tendency of essential genes to be more evolutionarily conserved may indeed explain the tendency of multifunctional genes to be more evolutionarily conserved.

2 Methods

Comparison of multifunctional and other annotated genes with correction for a gene feature

Comparison of the set of multifunctional genes M and other annotated genes N with correction for a gene feature f—e.g., degree or the number of associated publications (one value for each gene)—is performed as follows. We sample with replacement $n = 1000$ times independently at random from the set of genes N, so that each sample s_i is the list of the same number of genes as M (potentially with repetitions) having the same distribution of the feature f as M. The procedure to select each sample s is as follows. Start with a list l of size $|M|$ of genes each of which is chosen from N uniformly at random. Then repeat the following: on each step try to swap a random element in l with a random gene from N, and accept the swap only if the distributions of f on l and M become more similar to each other, as measured by the Mann–Whitney U statistic. Continue with these steps until the relative change in the U statistic has been less than 10^{-4} in the past 100 steps (in practice this requires $< 10^5$ steps). The samples s_i, $i = 1, \ldots, n$ produced with this method are used for the corrected comparison of M and N.

For a corrected comparison of M and N with respect to a certain gene property D—e.g., association with disease (given as a set of genes having the property)—compute the frequency x of D in M and frequencies y_i of D in samples s_i. Then, estimate the corrected frequency of D in N as the median of all y_i. Use the distribution of y_i to compute the 95% confidence interval (using 2.5% quantile cutoffs on both sides) and the empirical p-value (as the fraction of times x is higher than y_i). Similarly, for the comparison with respect to a gene feature B—e.g., betweenness centrality (one value for each gene)—compute the distribution of B in M, including the median b. Also, compute the distributions of B in each sample s_i, including the medians b_i. Then, estimate the corrected median of B in N as the median of all b_i. Also, use the distribution of b_i to compute the 95% confidence interval (using 2.5% quantile cutoffs on both sides) and the empirical p-value (as the fraction of times b is higher than b_i). Use the distributions of B in samples s_i all merged together to compute 25% and 75% quantiles.
