# Supplementary figures and images for: Genome-Wide Detection and Analysis of Multifunctional Genes
Source: PLoS Comput Biol. 2015 Oct 5;11(10):e1004467. doi: 10.1371/journal.pcbi.1004467 (PMC4593560; doi:10.1371/journal.pcbi.1004467)

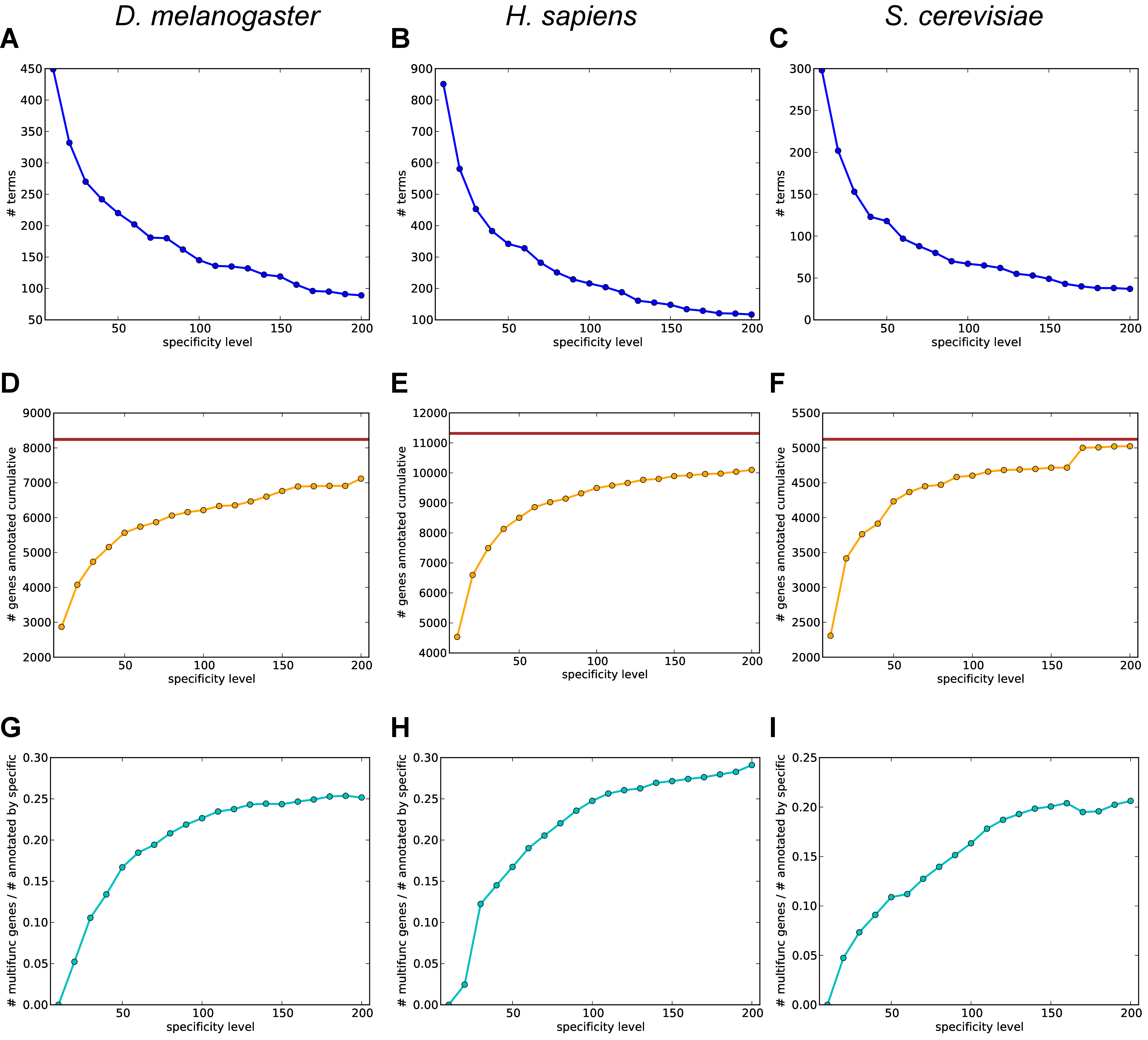

Supplement: S1 Fig — (A-C) Terms chosen at different specificity levels. The number of Biological Process (BP) Gene Ontology (GO) terms chosen is shown for each specificity threshold from 10 to 200 (increment of 10) for (A) fly, (B) human, (C) yeast. (D-F) Genes annotated with terms chosen at different specificity levels. For each M from 10 to 200 (increment of 10), the cumulative number of genes annotated with terms chosen for specificity thresholds N ≤ M is shown for (D) fly, (E) human, (F) yeast. Horizontal line shows the total number of genes annotated with any BP term. (G-I) Fraction of multifunctional genes in all annotated genes. For each specificity threshold, the fraction of the cumulative number of multifunctional genes to the total number of all genes annotated with terms chosen at this threshold is shown for (G) fly, (H) human, (I) yeast. See Materials and Methods for details. (PNG) [file pcbi.1004467.s002.png]

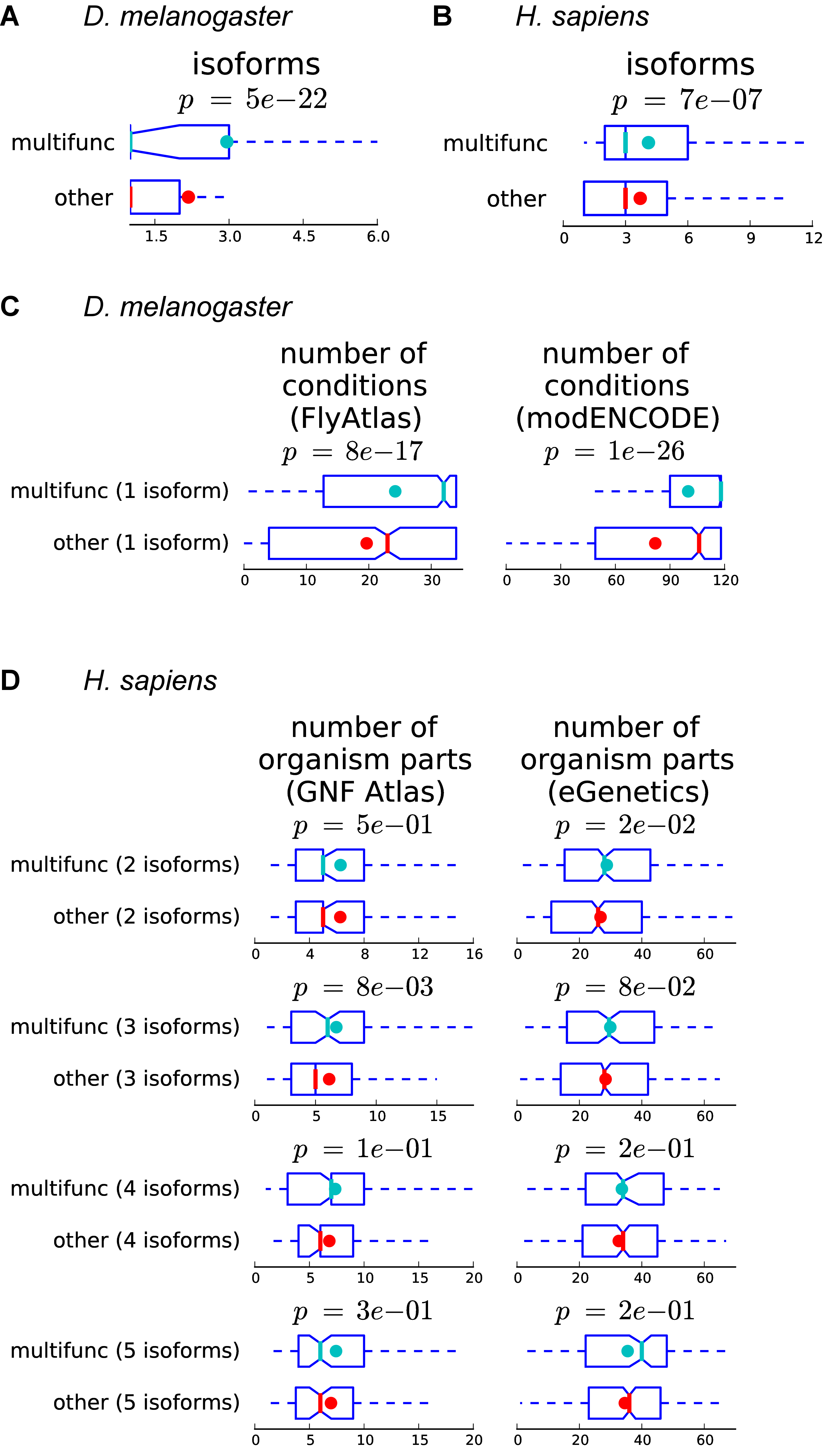

Supplement: S2 Fig — Boxplots of the number of isoforms per gene for multifunctional and other annotated genes in (A) fly and (B) human. Multifunctional genes have significantly larger number of isoforms (Mann–Whitney U). (C) Boxplots of the number of conditions in which multifunctional and other annotated genes in fly are expressed, for genes with one isoform only (which constitute 49% of multifunctional genes and 59% of other annotated genes). (D) Boxplots of the number of organism parts in which multifunctional and other annotated genes in human are expressed, for genes with 2 to 5 isoforms (17% of multifunctional and 18% of other genes have 2 isoforms, 14% of multifunctional and 14% of other genes have 3 isoforms, 10% of multifunctional and 11% of other genes have 4 isoforms, 9% of multifunctional and 8% of other genes have 5 isoforms). See Fig 3 for comparison across all genes. (PNG) [file pcbi.1004467.s003.png]

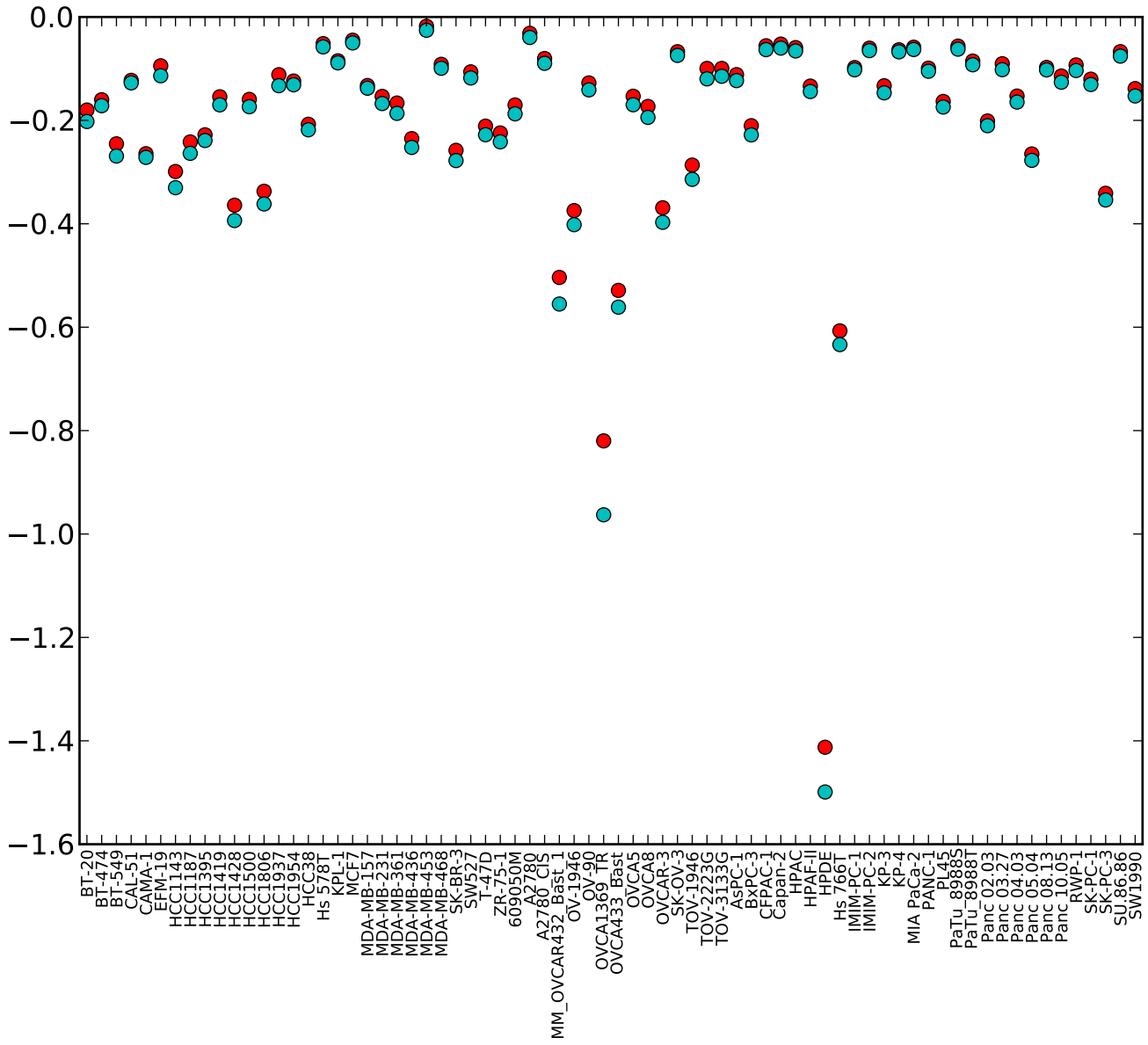

Supplement: S3 Fig — For each of 72 human cancer cell lines (x-axis) in the COLT-Cancer database [43, 44], the median GARP score of essentiality, as reported in the database, is shown for multifunctional (cyan) and all other annotated (red) genes on the y-axis; lower GARP scores depict higher essentiality. Multifunctional genes tend to be more essential than other annotated genes in all 72 cell lines. (PDF) [file pcbi.1004467.s004.pdf]

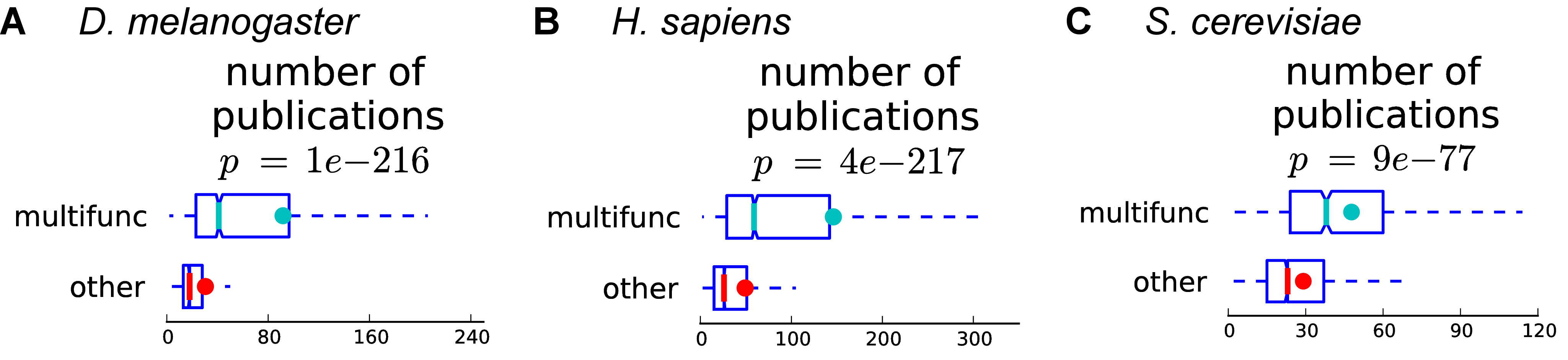

Supplement: S4 Fig — Boxplots of the number of PubMed publications associated with multifunctional and other annotated genes are shown for (A) fly, (B) human, and (C) yeast. Multifunctional genes are associated with a significantly larger number of publications (Mann–Whitney U test). (PNG) [file pcbi.1004467.s005.png]

with disease

$$p = 0.009$$

multifunc

other

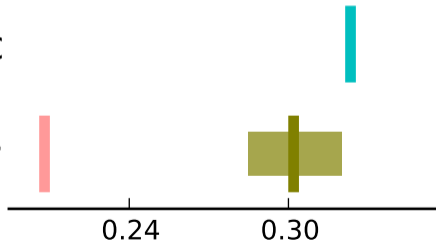

with two diseases

$$p < 0.001$$

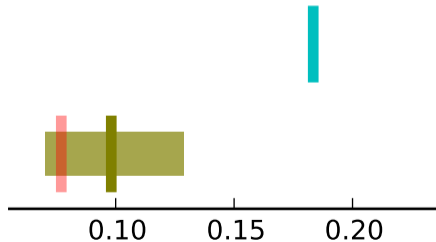

Supplement: S5 Fig — Fractions of multifunctional (cyan) and other annotated (red) genes associated with diseases are shown (same as in Fig 7), as well as the estimated fractions in other genes after controlling for study bias (olive, with the boxes giving the 95% confidence intervals). The estimation is from 1000 independent random samples from the set of other annotated genes, where the samples have the same distribution of the number of associated publications as multifunctional genes. Multifunctional genes are associated with significantly larger number of diseases even after controlling for study bias (empirical p-values shown). See Methods in S1 Text for details. (PDF) [file pcbi.1004467.s006.pdf]

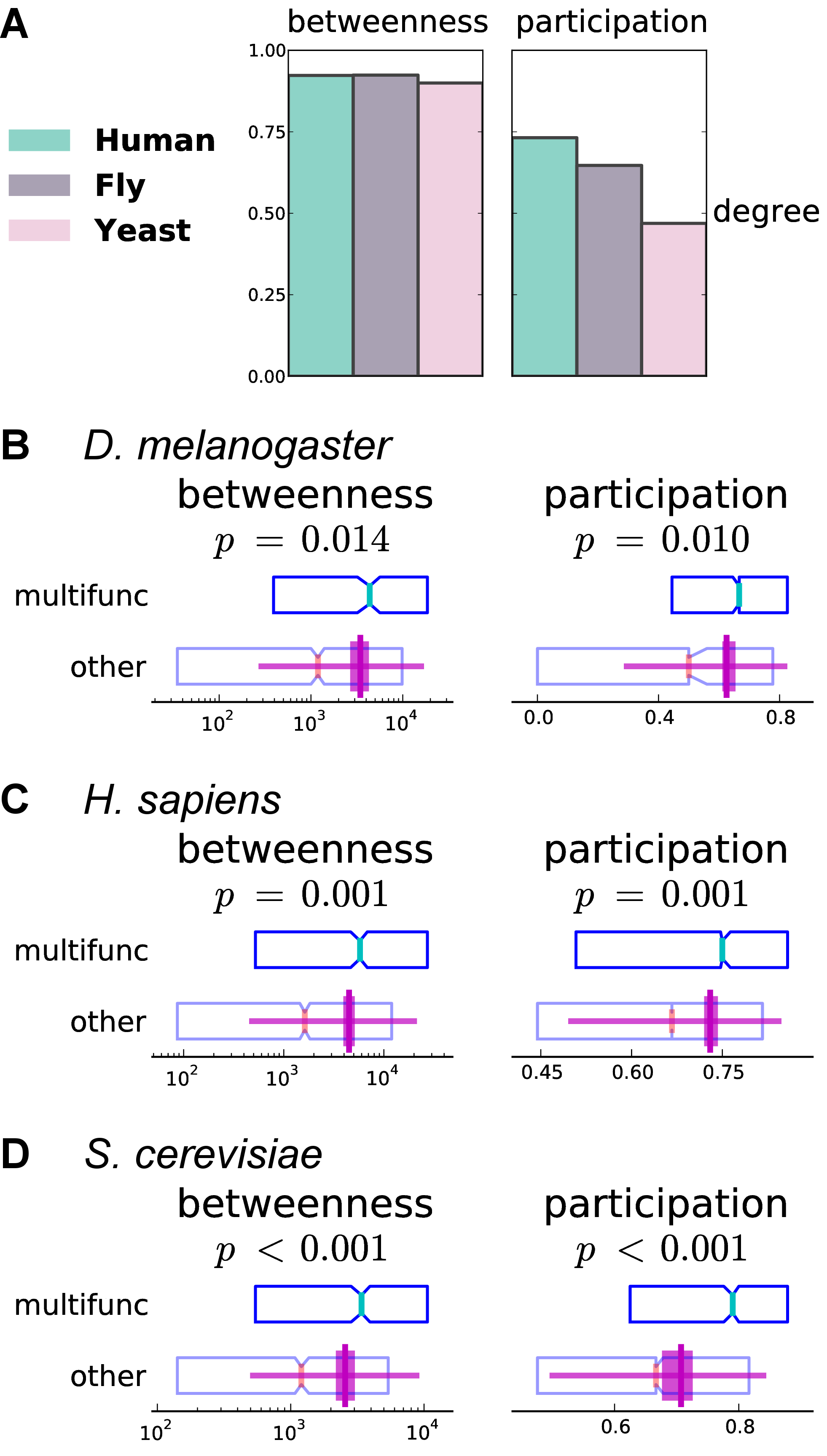

Supplement: S6 Fig — (A) Barplots of the Spearman correlations between degree and betweenness centrality and participation coefficient, as measured for fly, human and yeast physical protein-protein interaction networks. Degree is highly correlated with both measures. (B–D) Comparison of betweenness and participation of multifunctional and other annotated genes, while controlling for degree. Boxplots show the distribution of betweenness or participation for multifunctional and other annotated genes for (B) fly, (C) human, (D) yeast (same as in Fig 8). In magenta are the distributions of the same measures for random samples from the set of other annotated genes, where the samples have the same degree distribution as multifunctional genes. Vertical magenta lines show the estimated medians, boxes show the 95% confidence intervals around the medians, and horizontal lines show the 25%–75% quantile ranges. After controlling for degree, the betweenness and participation of multifunctional genes are significantly higher than for other annotated genes (empirical p-value computed for comparing medians). See Methods in S1 Text for details. (PNG) [file pcbi.1004467.s007.png]

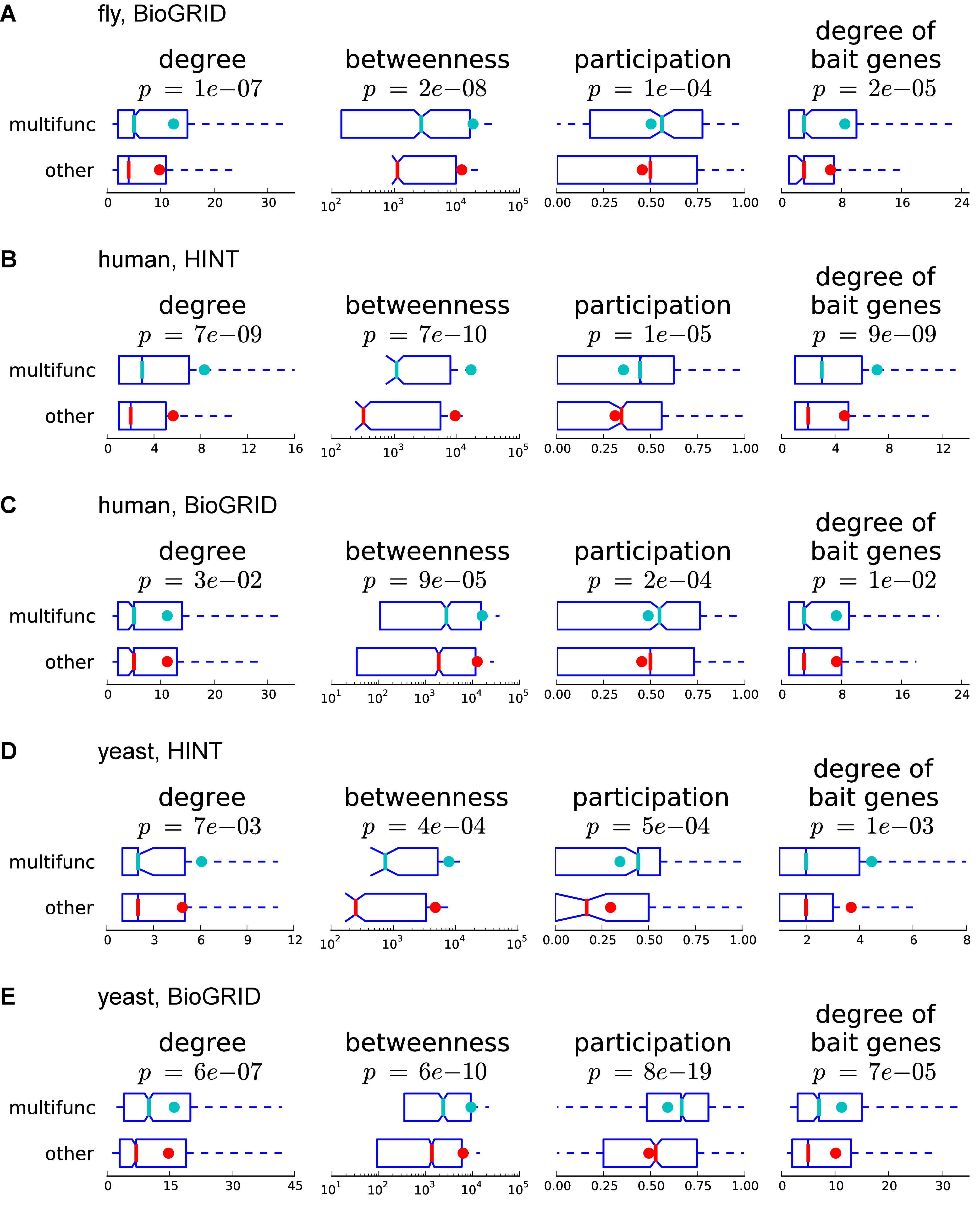

Supplement: S7 Fig — Boxplots with three measures of centrality—degree (number of interactions), betweenness centrality, and participation coefficient—in high-throughput protein interaction networks. Comparisons of multifunctional and other annotated genes are shown for (A) fly (BioGRID), (B) human (HINT), (C) human (BioGRID), (D) yeast (HINT), (E) yeast (BioGRID). Multifunctional genes are significantly more central than other annotated genes (Mann–Whitney U test) in high-throughput networks that are not prone to bias towards more studied genes. For an even stricter comparison, the degree of bait genes—i.e., the number of interactions from bait to prey genes in these high-throughput experiments—is compared between multifunctional and all other annotated genes, and the trend is confirmed in all networks (A–E). (PNG) [file pcbi.1004467.s008.png]

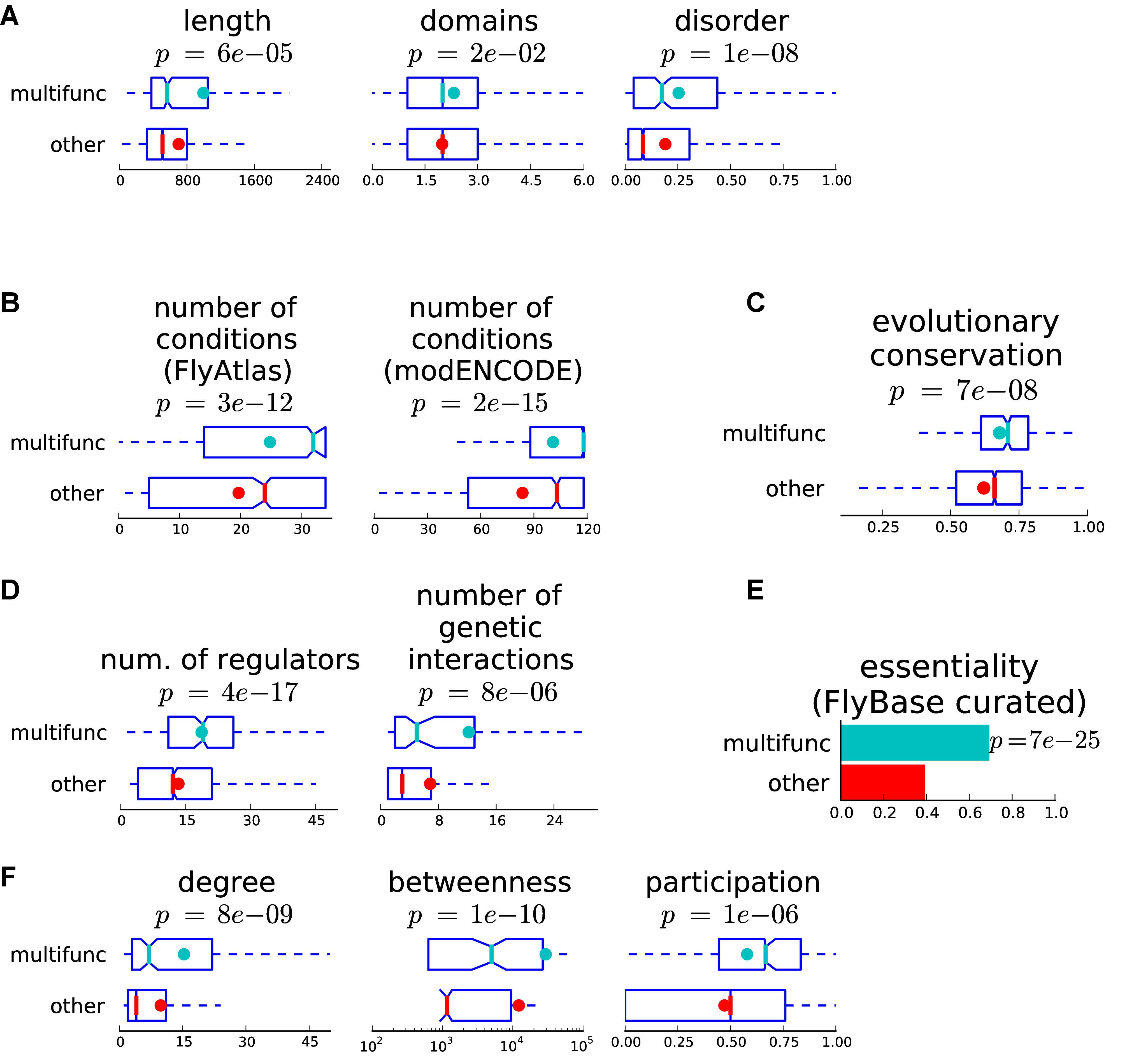

Supplement: S8 Fig — Comparison of multifunctional and other annotated genes obtained from the Molecular Function Gene Ontology using our method (see Fig 1 and Materials and Methods). (A) Physicochemical properties (compare with Fig 2A). (B) Expression (compare with Fig 3A). (C) Evolutionary conservation (compare with Fig 4A). (D) Regulatory and genetic interactions (compare with Fig 5A). (E) Essentiality (FlyBase curated; compare with Fig 6A). (F) Centrality in protein-protein interaction networks (compare with Fig 8A). (PNG) [file pcbi.1004467.s009.png]

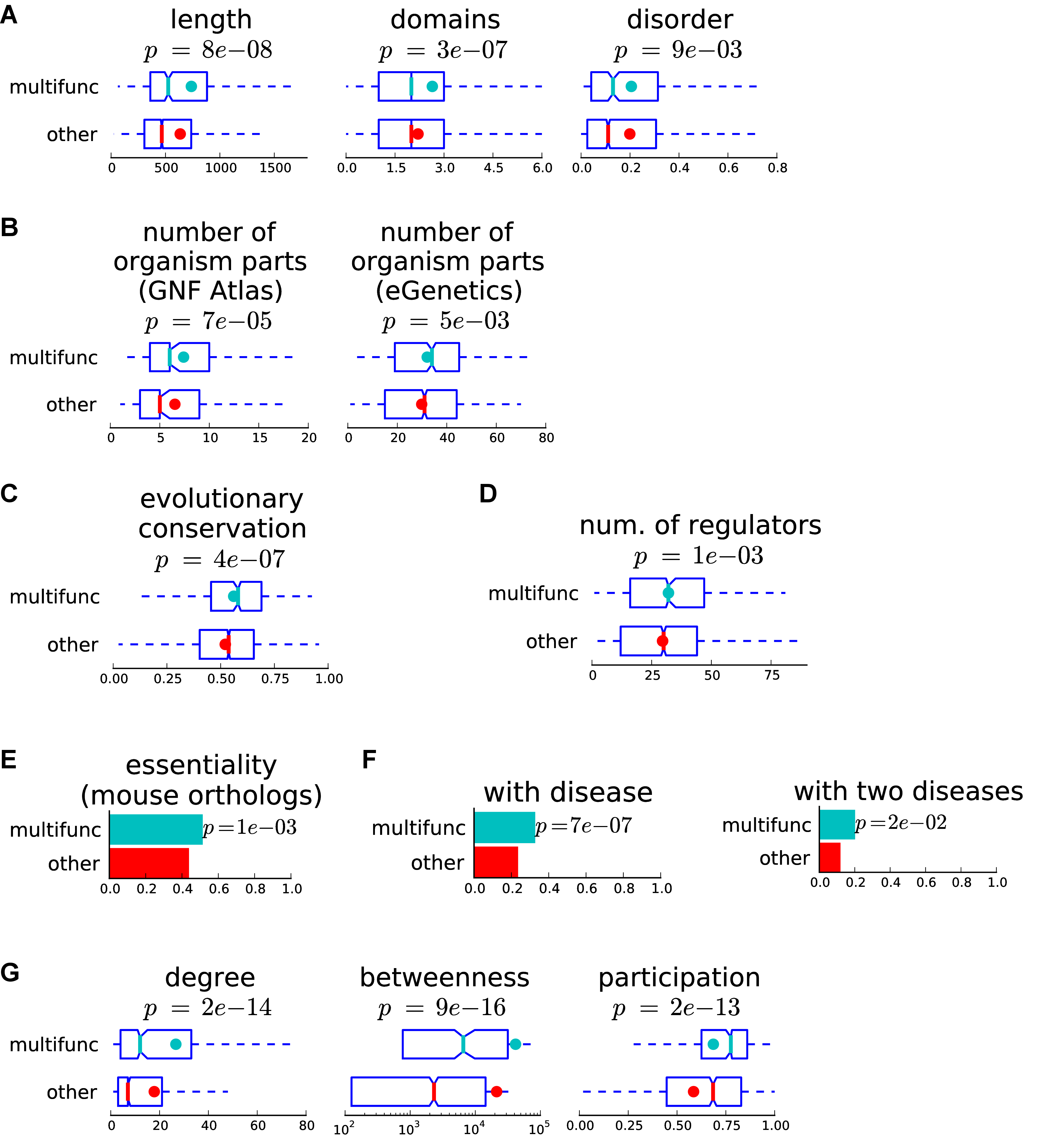

Supplement: S9 Fig — Comparison of multifunctional and other annotated genes obtained from the Molecular Function Gene Ontology using our method (see Fig 1 and Materials and Methods). (A) Physicochemical properties (compare with Fig 2B). (B) Expression (compare with Fig 3B). (C) Evolutionary conservation (compare with Fig 4B). (D) Regulatory interactions (compare with Fig 5B). (E) Essentiality (mouse orthologs; compare with Fig 6C). (F) Association with diseases (compare with Fig 7). (G) Centrality in protein-protein interaction networks (compare with Fig 8B). (PNG) [file pcbi.1004467.s010.png]

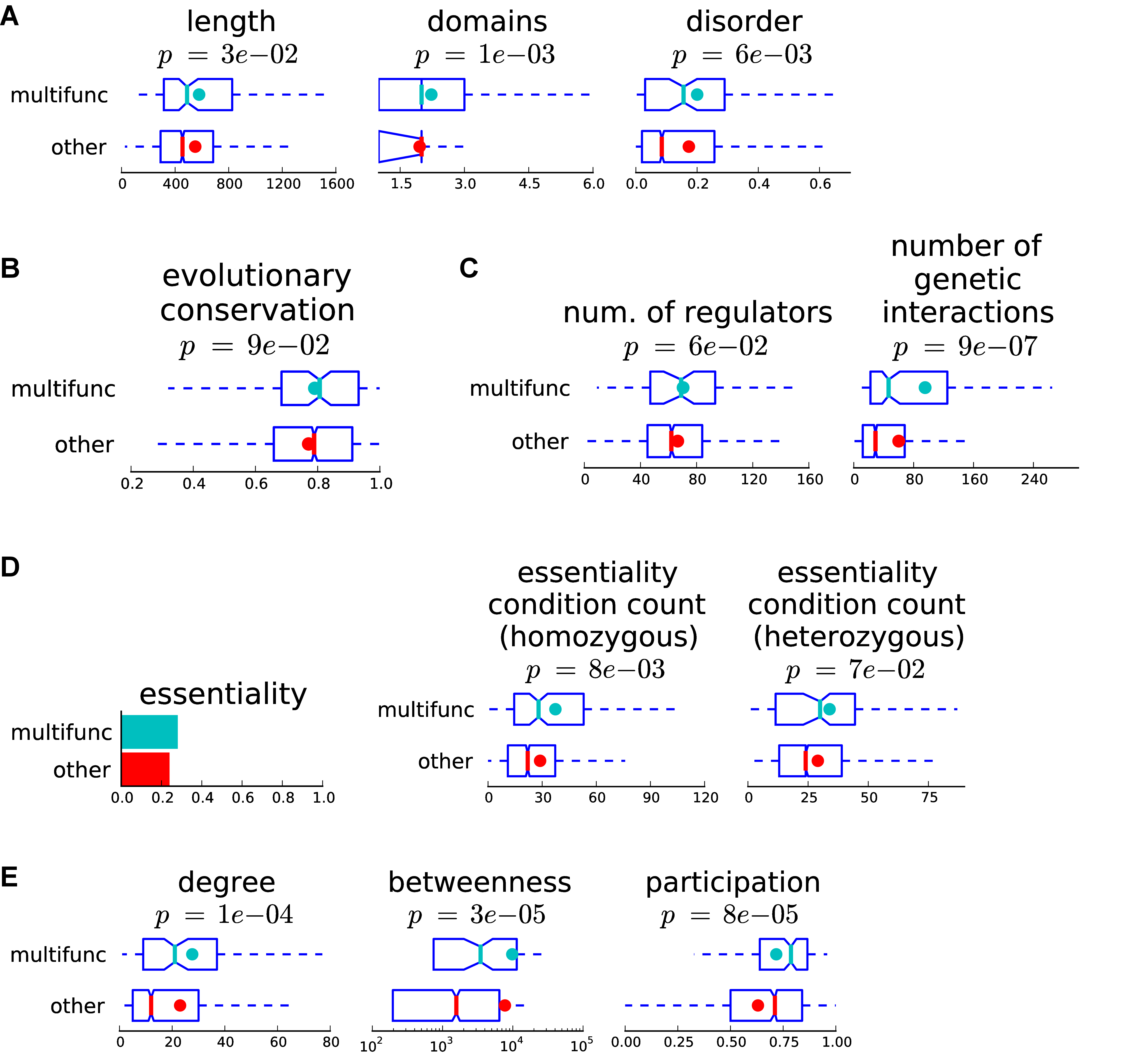

Supplement: S10 Fig — Comparison of multifunctional and other annotated genes obtained from the Molecular Function Gene Ontology using our method (see Fig 1 and Materials and Methods). (A) Physicochemical properties (compare with Fig 2C). (B) Evolutionary conservation (compare with Fig 4C). (C) Regulatory and genetic interactions (compare with Fig 5C). (D) Essentiality (compare with Fig 6E and 6F). (E) Centrality in protein-protein interaction networks (compare with Fig 8C). (PNG) [file pcbi.1004467.s011.png]

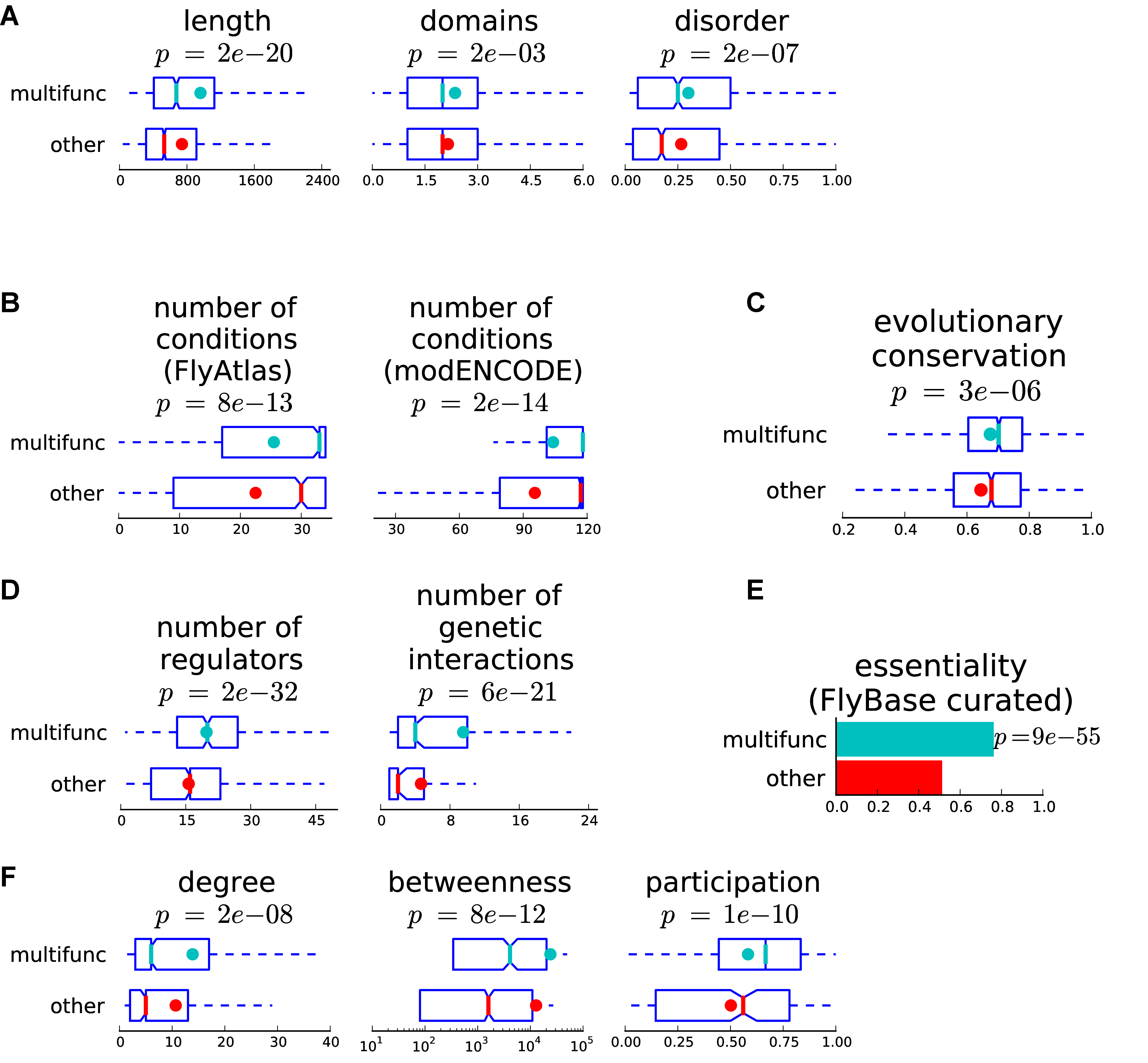

Supplement: S11 Fig — Comparison of multifunctional and other annotated genes obtained from the most reliable GO BP annotations (with evidence codes EXP, IDA, IMP, IEP, IC, TAS) using our method (see Fig 1 and Materials and Methods). (A) Physicochemical properties (compare with Fig 2A). (B) Expression (compare with Fig 3A). (C) Evolutionary conservation (compare with Fig 4A). (D) Regulatory and genetic interactions (compare with Fig 5A). (E) Essentiality (FlyBase curated; compare with Fig 6A). (F) Centrality in protein-protein interaction networks (compare with Fig 8A). (PNG) [file pcbi.1004467.s012.png]

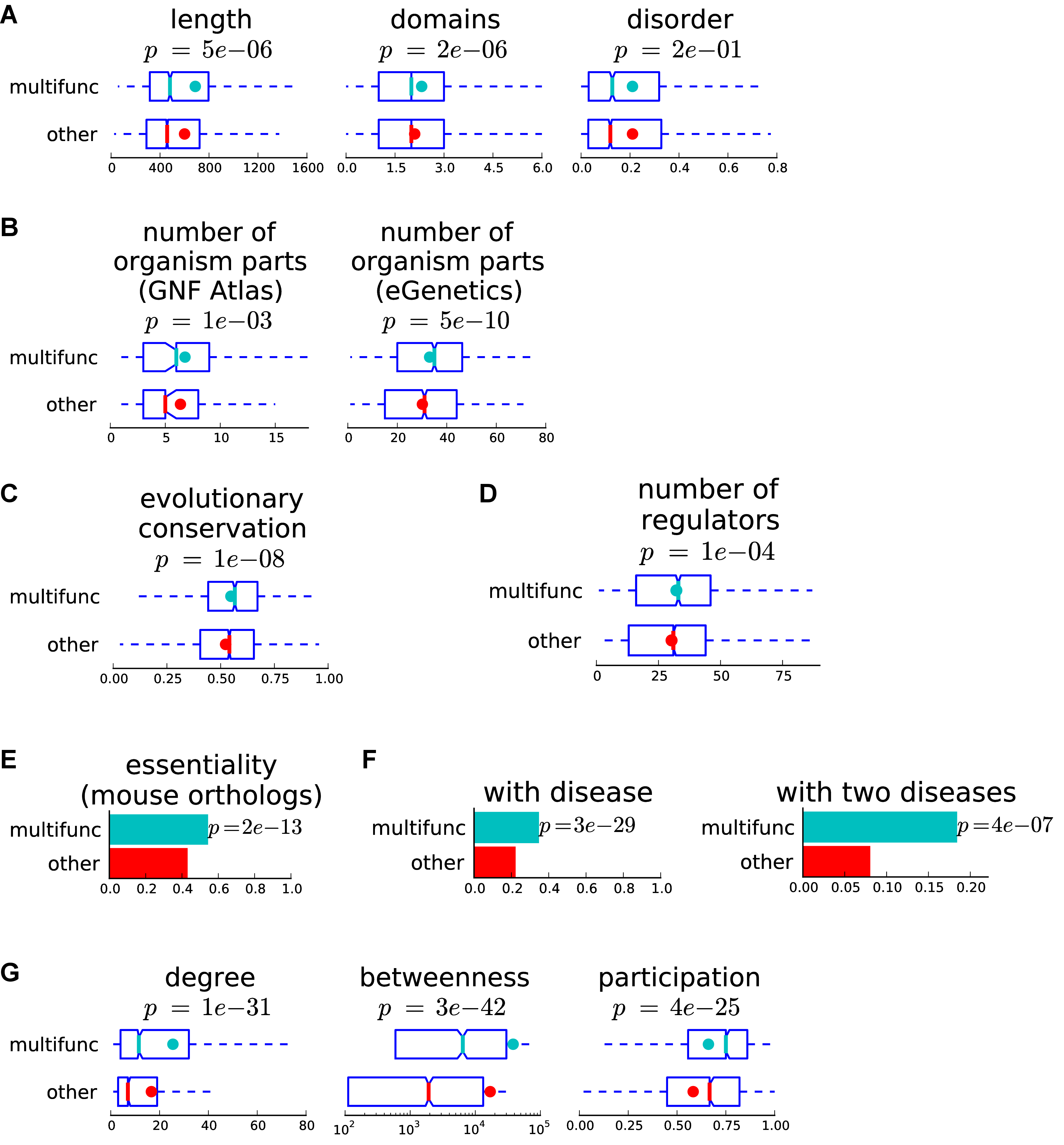

Supplement: S12 Fig — Comparison of multifunctional and other annotated genes obtained from the most reliable GO BP annotations (with evidence codes EXP, IDA, IMP, IEP, IC, TAS) using our method (see Fig 1 and Materials and Methods). (A) Physicochemical properties (compare with Fig 2B). (B) Expression (compare with Fig 3B). (C) Evolutionary conservation (compare with Fig 4B). (D) Regulatory interactions (compare with Fig 5B). (E) Essentiality (mouse orthologs; compare with Fig 6C). (F) Association with diseases (compare with Fig 7). (G) Centrality in protein-protein interaction networks (compare with Fig 8B). (PNG) [file pcbi.1004467.s013.png]

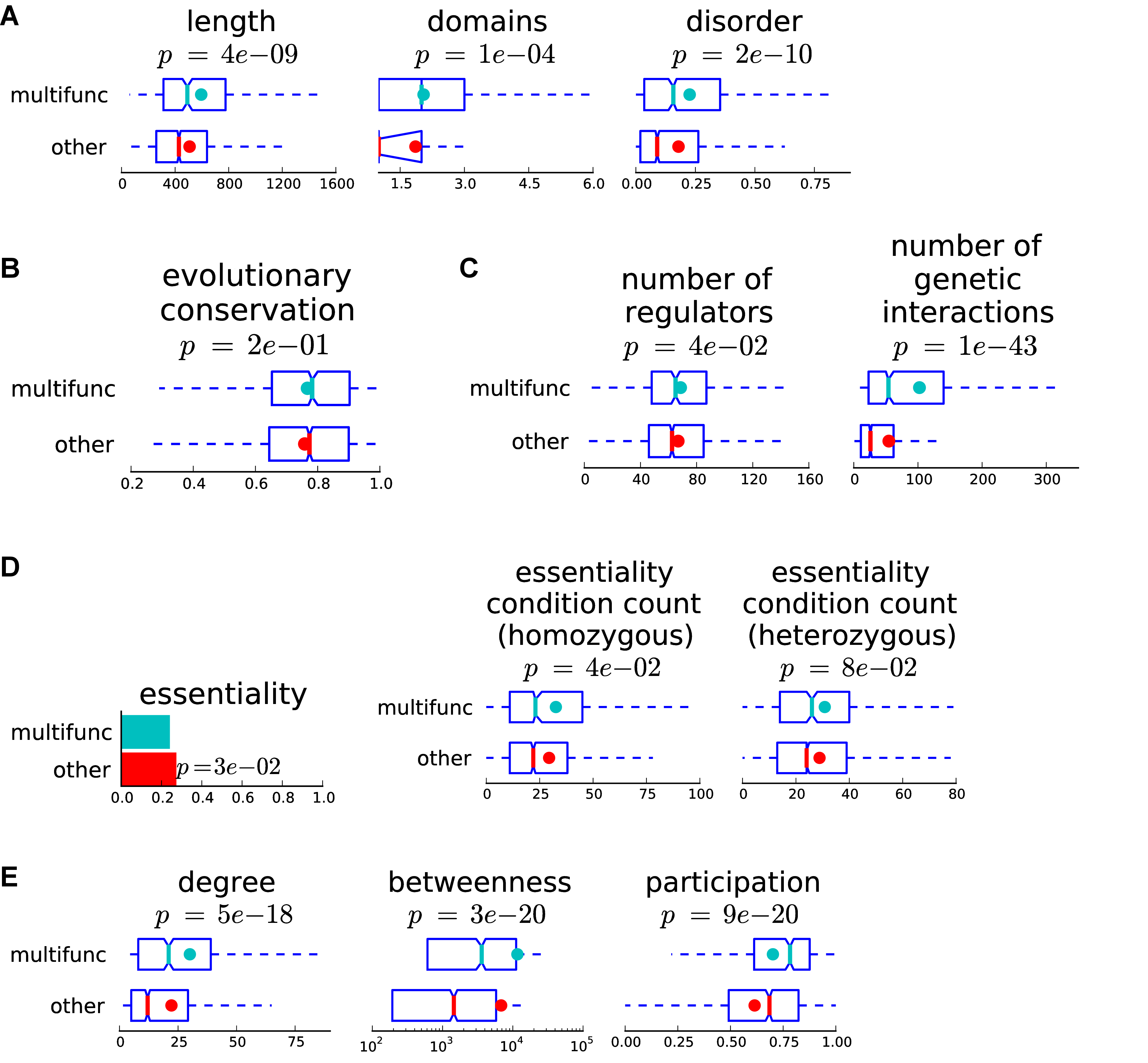

Supplement: S13 Fig — Comparison of multifunctional and other annotated genes obtained from the most reliable GO BP annotations (with evidence codes EXP, IDA, IMP, IEP, IC, TAS) using our method (see Fig 1 and Materials and Methods). (A) Physicochemical properties (compare with Fig 2C). (B) Evolutionary conservation (compare with Fig 4C). (C) Regulatory and genetic interactions (compare with Fig 5C). (D) Essentiality (compare with Fig 6E and 6F). (E) Centrality in protein-protein interaction networks (compare with Fig 8C). (PNG) [file pcbi.1004467.s014.png]

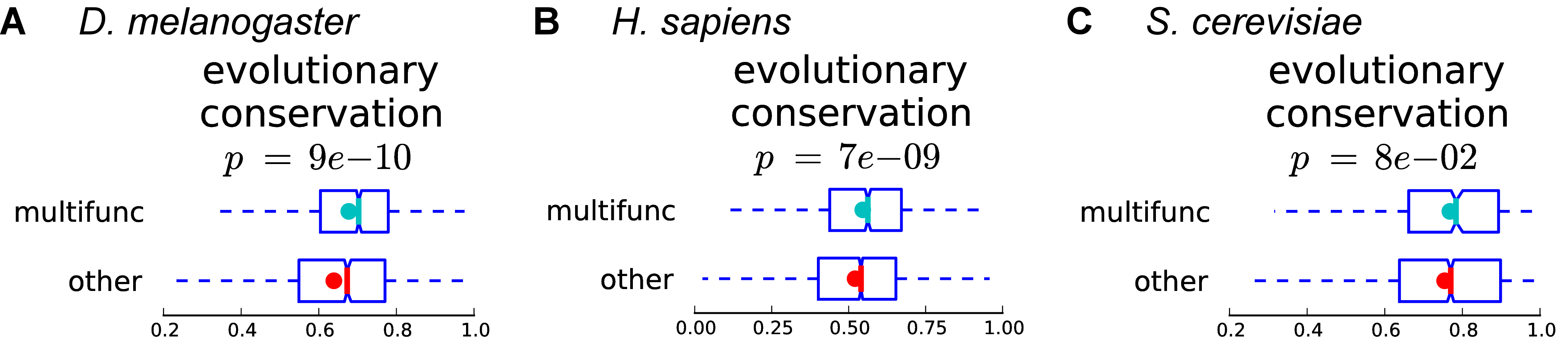

Supplement: S14 Fig — Boxplots of evolutionary conservation (estimated by phastCons [32] for each nucleotide, averaged over the nucleotides of each gene) of multifunctional and other annotated genes are shown for (A) fly, (B) human, and (C) yeast. When detecting multifunctional genes, from the set of annotations used in the analysis in the main text (see Materials and Methods), those with evidence codes ISS, ISA, ISO, ISM are removed. Colored dots show means, notches show bootstrap-generated 95% confidence intervals around the medians, boxes show quartile ranges, and whiskers extend to the most extreme data points within 1.5 times the size of the inner quartile range. Multifunctional genes are significantly more evolutionary conserved than other genes (Mann–Whitney U test). Compare with Fig 4. (PNG) [file pcbi.1004467.s015.png]
