# Supplementary material for: Genome-Wide Detection and Analysis of Multifunctional Genes
Source: PLoS Comput Biol. 2015 Oct 5;11(10):e1004467. doi: 10.1371/journal.pcbi.1004467 (PMC4593560; doi:10.1371/journal.pcbi.1004467)
Supplement: S1 Table — Spearman correlations (with p-values) between the number of isoforms of a gene and the number of tissues or organism parts in which the gene is expressed, according to genome-wide assays in fly and human (see main text and Materials and Methods). (PDF) [file pcbi.1004467.s016.pdf]

S1 Table

Genes with more isoforms tend to be detected as more broadly expressed. Spearman correlations (with p-values) between the number of isoforms of a gene and the number of tissues or organism parts in which the gene is expressed, according to genome-wide assays in fly and human (see main text and **Materials and methods**).

expression assay	Spearman's ρ
D. melanogaster	
FlyAtlas	0.18 ($p < 5e{-}61$)
modENCODE	0.20 ($p < 3e{-}80$)
H. sapiens	
GNF Atlas	0.12 ($p < 5e{-}34$)
eGenetics	0.33 ($p < 5e{-}238$)
