# Supplementary material for: Genome-Wide Detection and Analysis of Multifunctional Genes
Source: PLoS Comput Biol. 2015 Oct 5;11(10):e1004467. doi: 10.1371/journal.pcbi.1004467 (PMC4593560; doi:10.1371/journal.pcbi.1004467)
Supplement: S2 Table — In the first part of the table, we show Spearman correlations (with p-values) between whether a gene is multifunctional (1 or 0 depending on whether it is found to be multifunctional or not) and its degree, betweenness, and participation in protein-protein interaction networks. All correlations are positive and significant (compare with Fig 8). In the second part of the table, we show partial Spearman correlations between gene multifunctionality and betweenness and participation, when controlling for degree. All partial correlations are small but positive, and are statistically significant (compare with S6 Fig). (PDF) [file pcbi.1004467.s017.pdf]

S2 Table

Multifunctionality and centrality in protein-protein physical interaction networks. In the first part of the table, we show Spearman correlations (with p-values) between whether a gene is multifunctional (1 or 0 depending on whether it is found to be multifunctional or not) and its degree, betweenness, and participation in protein-protein interaction networks. All correlations are positive and significant (compare with Fig 8). In the second part of the table, we show partial Spearman correlations between gene multifunctionality and betweenness and participation, when controlling for degree. All partial correlations are small but positive, and are statistically significant (compare with S6 Fig).

correlation with multifunctionality			
organism	degree	betweenness	participation
fly	0.13 ($p < 1e{-}15$)	0.15 ($p < 1e{-}19$)	0.12 ($p < 3e{-}13$)
human	0.15 ($p < 1e{-}36$)	0.17 ($p < 1e{-}50$)	0.13 ($p < 6e{-}31$)
yeast	0.15 ($p < 2e{-}21$)	0.16 ($p < 6e{-}26$)	0.18 ($p < 1e{-}31$)

partial correlation with multifunctionality corrected for degree			
organism		betweenness	participation
fly		0.07 ($p < 2e{-}5$)	0.05 ($p < 2e{-}3$)
human		0.10 ($p < 3e{-}17$)	0.05 ($p < 4e{-}5$)
yeast		0.07 ($p < 6e{-}6$)	0.13 ($p < 4e{-}18$)
