# Supplementary material for: Genome-Wide Detection and Analysis of Multifunctional Genes
Source: PLoS Comput Biol. 2015 Oct 5;11(10):e1004467. doi: 10.1371/journal.pcbi.1004467 (PMC4593560; doi:10.1371/journal.pcbi.1004467)
Supplement: S3 Table — Analysis of multifunctional genes derived from the Biological Process ontology (BP-multifunctional) using the specificity parameter upper bound 120 (the same as used in the main analysis of the paper; see Figs 2, 3, 4, 5, 6, 7, 8), when compared with multifunctional genes derived from the Molecular Function ontology (MF-multifunctional) using the specificity parameter upper bounds 120 (a more specific cut-off) and 500 (a more general cut-off allowing more genes to be detected as MF-multifunctional). For each organism, shown is the number of BP-multifunctional genes (see Table 1); the number of them annotated with specific terms from MF; the number and percent of such genes that are detected as MF-multifunctional; and the p-value from the hypergeometric test corresponding to the significance of this intersection. A significant fraction of BP-multifunctional genes are also MF-multifunctional. (PDF) [file pcbi.1004467.s018.pdf]

## S3 Table

**Comparison of BP-multifunctional to MF-multifunctional genes.** Analysis of multifunctional genes derived from the Biological Process ontology (BP-multifunctional) using the specificity parameter upper bound 120 (the same as used in the main analysis of the paper; see Fig 2, Fig 3, Fig 4, Fig 5, Fig 6, Fig 7, Fig 8), when compared with multifunctional genes derived from the Molecular Function ontology (MF-multifunctional) using the specificity parameter upper bounds 120 (a more specific cut-off) and 500 (a more general cut-off allowing more genes to be detected as MF-multifunctional). For each organism, shown is the number of BP-multifunctional genes (see Table 1); the number of them annotated with specific terms from MF; the number and percent of such genes that are detected as MF-multifunctional; and the $p$-value from the hypergeometric test corresponding to the significance of this intersection. A significant fraction of BP-multifunctional genes are also MF-multifunctional.

| organism | BP-multifunctional | BP-multifunctional annotated in MF by terms used to detect MF-multifunctionality | BP-multifunctional and MF-multifunctional | % | $p$-value |
|---|---|---|---|---|---|
| MF specificity upper bound 120 | | | | | |
| *D. melanogaster* | 1509 | 1210 | 223 | 18% | $9e{-}59$ |
| *H. sapiens* | 2517 | 1967 | 390 | 20% | $9e{-}62$ |
| *S. cerevisiae* | 876 | 682 | 81 | 12% | $1e{-}21$ |
| MF specificity upper bound 500 | | | | | |
| *D. melanogaster* | 1509 | 1336 | 402 | 30% | $1e{-}87$ |
| *H. sapiens* | 2517 | 2168 | 760 | 35% | $1e{-}102$ |
| *S. cerevisiae* | 876 | 731 | 128 | 18% | $6e{-}18$ |
