# Supplementary material for: Genome-Wide Detection and Analysis of Multifunctional Genes
Source: PLoS Comput Biol. 2015 Oct 5;11(10):e1004467. doi: 10.1371/journal.pcbi.1004467 (PMC4593560; doi:10.1371/journal.pcbi.1004467)
Supplement: S4 Table — Analysis of multifunctional genes derived from the Molecular Function ontology (MF-multifunctional) using the specificity parameter upper bound 120 (used in the analysis shown in S8, S9, S10 Figs) when compared with multifunctional genes derived from the Biological Process ontology (BP-multifunctional) using the specificity parameter upper bounds 120 (a more specific cut-off) and 500 (a more general cut-off allowing more genes to be detected as BP-multifunctional). For each organism, shown is the number of MF-multifunctional genes; the number of them annotated with specific terms from BP; the number and percent of such genes that are detected as BP-multifunctional; and the p-value from the hypergeometric test corresponding to the significance of intersection. Most MF-multifunctional genes are also BP-multifunctional. (PDF) [file pcbi.1004467.s019.pdf]

S4 Table

Comparison of MF-multifunctional to BP-multifunctional genes. Analysis of multifunctional genes derived from the Molecular Function ontology (MF-multifunctional) using the specificity parameter upper bound 120 (used in the analysis shown in S8 Fig, S9 Fig, S10 Fig) when compared with multifunctional genes derived from the Biological Process ontology (BP-multifunctional) using the specificity parameter upper bounds 120 (a more specific cut-off) and 500 (a more general cut-off allowing more genes to be detected as BP-multifunctional). For each organism, shown is the number of MF-multifunctional genes; the number of them annotated with specific terms from BP; the number and percent of such genes that are detected as BP-multifunctional; and the *p*-value from the hypergeometric test corresponding to the significance of intersection. Most MF-multifunctional genes are also BP-multifunctional.

organism	MF-multifunctional	MF-multifunctional annotated in BP by terms used to detect BP-multifinctionality	MF-multifunctional and BP-multifunctional	%	*p*-value
BP specificity upper bound 120					
D. melanogaster	324	309	223	72%	$9e{-}59$
H. sapiens	607	584	390	67%	$9e{-}62$
S. cerevisiae	149	144	81	56%	$1e{-}21$
BP specificity upper bound 500					
D. melanogaster	324	316	248	78%	$2e{-}68$
H. sapiens	607	600	458	76%	$3e{-}61$
S. cerevisiae	149	145	101	70%	$2e{-}26$
